# T follicular helper cells contribute to pathophysiology in a model of neuromyelitis optica spectrum disorders

**DOI:** 10.1172/jci.insight.161003

**Published:** 2023-02-22

**Authors:** Leung-Wah Yick, Oscar Ka-Fai Ma, Ethel Yin-Ying Chan, Krystal Xiwing Yau, Jason Shing-Cheong Kwan, Koon-Ho Chan

**Affiliations:** Department of Medicine and Neuroimmunology and Neuroinflammation Research Laboratory, Li Ka Shing Faculty of Medicine, The University of Hong Kong, Hong Kong, China.

**Keywords:** Autoimmunity, Neuroscience, Autoimmune diseases, Demyelinating disorders, T cells

## Abstract

Neuromyelitis optica spectrum disorders (NMOSD) are inflammatory autoimmune disorders of the CNS. IgG autoantibodies targeting the aquaporin-4 water channel (AQP4-IgGs) are the pathogenic effector of NMOSD. Dysregulated T follicular helper (Tfh) cells have been implicated in loss of B cell tolerance in autoimmune diseases. The contribution of Tfh cells to disease activity and therapeutic potential of targeting these cells in NMOSD remain unclear. Here, we established an autoimmune model of NMOSD by immunizing mice against AQP4 via in vivo electroporation. After AQP4 immunization, mice displayed AQP4 autoantibodies in blood circulation, blood-brain barrier disruption, and IgG infiltration in spinal cord parenchyma. Moreover, AQP4 immunization induced motor impairments and NMOSD-like pathologies, including astrocytopathy, demyelination, axonal loss, and microglia activation. These were associated with increased splenic Tfh, Th1, and Th17 cells; memory B cells; and plasma cells. *Aqp4*-deficient mice did not display motor impairments and NMOSD-like pathologies after AQP4 immunization. Importantly, abrogating ICOS/ICOS-L signaling using anti–ICOS-L antibody depleted Tfh cells and suppressed the response of Th1 and Th17 cells, memory B cells, and plasma cells in AQP4-immunized mice. These findings were associated with ameliorated motor impairments and spinal cord pathologies. This study suggests a role of Tfh cells in the pathophysiology of NMOSD in a mouse model with AQP4 autoimmunity and provides an animal model for investigating the immunological mechanisms underlying AQP4 autoimmunity and developing therapeutic interventions targeting autoimmune reactions in NMOSD.

## Graphical abstract

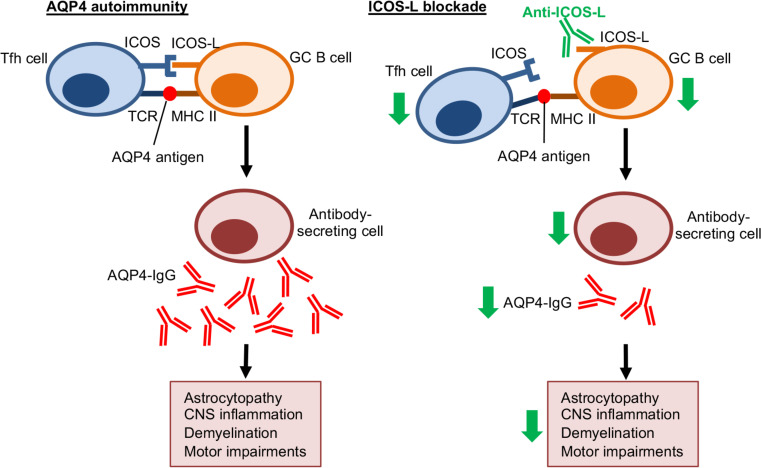

## Introduction

Neuromyelitis optica spectrum disorders (NMOSD) are severe inflammatory demyelinating disorders of the CNS clinically characterized by optic neuritis, myelitis, and encephalitis (1). Most patients with NMOSD are seropositive for IgG autoantibodies targeting the water channel aquaporin-4 (AQP4) (2, 3), which is highly expressed on the membrane of astrocyte end feet. AQP4-IgGs are directly pathogenic, and patients with NMOSD who are seropositive for these autoantibodies have underlying AQP4 autoimmunity (4). Less is known about the immunological mechanisms underlying AQP4-IgG production, although loss of B cell tolerance has been suggested to be a contributing factor. In the CNS, the binding of AQP4-IgG to astrocytic AQP4 leads to the development of NMOSD pathologies including astrocytopathy, glutamate excitotoxicity, blood-brain barrier (BBB) disruption, neuroinflammation, demyelination, and neuronal injury (5–10).

The generation of AQP4-specific autoreactive B cells has been suggested to be a consequence of impaired immunological tolerance (11). In an ex vivo study of B cell subsets derived from patients with NMOSD, the production of B cells secreting AQP4-specific antibody was promoted by a culture condition that mimicked T cell help (12). T follicular helper (Tfh) cells are critical for the differentiation of B cells into memory B cells, plasmablasts, and plasma cells that produce isotype-switched, high-affinity antibodies (13). They are characterized by high-level expression of CXC chemokine receptor type 5 (CXCR5), programmed cell death protein 1 (PD-1), and inducible costimulator (ICOS) (13).

Tfh cell differentiation occurs in secondary lymphoid organs including lymph nodes and the spleen. In the T cell zone, dendritic cells present antigen and costimulatory signals to naive Th cells. Then pre-Tfh cells migrate to the T-B cell border, where they receive antigen presentation and ICOS ligand (ICOS-L) costimulation from antigen-specific B cells. Fully differentiated Tfh cells migrate to germinal centers (GCs). They form cognate interactions with GC B cells, and secrete interleukin-21 (IL-21), IL-4, CD40L, and CXCL13 to support B cell survival and differentiation into antibody-secreting cells. Notably, sustained ICOS/ICOS-L signaling is needed for early T cell activation and Tfh cell differentiation and migration, as well as Tfh cell phenotype maintenance. The idea of disrupting the interaction between Tfh cells and B cells to remove potentially pathogenic B cells has received significant attention in the field of autoimmune diseases (14). Recent studies have found a correlation between the expansion of circulating Tfh cells and the disease activity in patients with NMOSD (15–18). However, the contribution of Tfh cells to immunopathogenesis and the therapeutic potential of targeting the ICOS/ICOS-L signaling pathway in NMOSD remain unclear.

Reported animal models of NMOSD are based on passive transfer of purified IgG from AQP4-IgG–seropositive patients or recombinant AQP4 antibodies to rodents (19–26). We recently showed that intraperitoneal injection of human AQP4-IgG induced motor impairments and NMOSD-like CNS pathologies in mice (27, 28). While these models are able to demonstrate the pathological effects of AQP4-IgG, they fail to recapitulate the autoimmunity underlying NMOSD with production of AQP4 autoantibodies. Here, we developed a mouse model of NMOSD with AQP4 autoimmunity using electroporation-mediated immunization. These mice displayed the production of IgG autoantibodies against AQP4 in blood circulation, and this was associated with motor impairments and NMOSD-like CNS pathologies. Importantly, the disease activity of these mice was reversed by suppression of Tfh cell response via blockade of the ICOS/ICOS-L signaling pathway.

## Results

### In vivo electroporation induces AQP4 overexpression in skeletal muscle.

**Figure 1:**
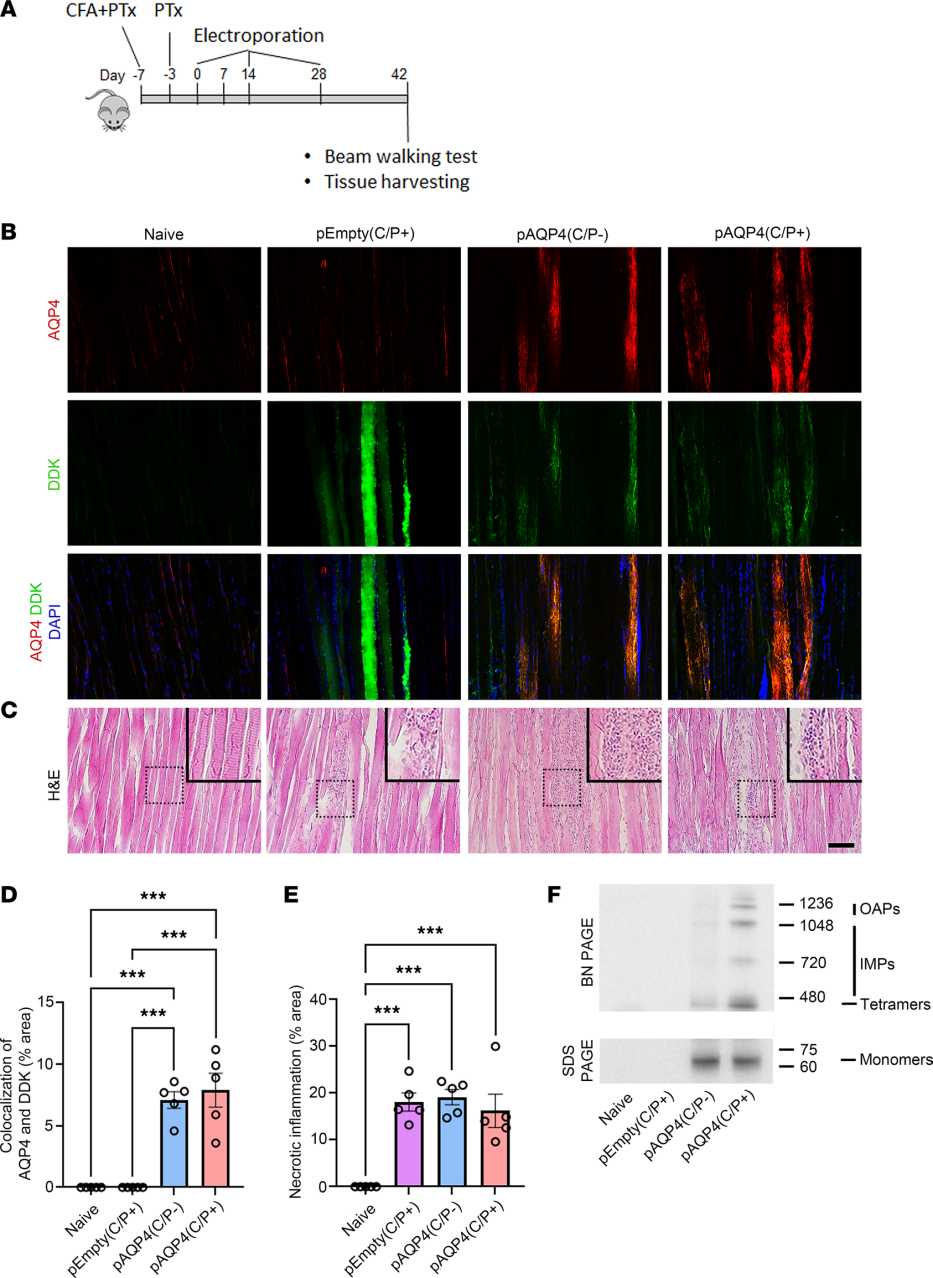
In vivo electroporation triggers AQP4 overexpression in skeletal muscle. (**A**) Experimental design. Mice were pretreated with CFA and PTx. Then animals received in vivo electroporation of pAQP4 or pEmpty at the left tibialis anterior muscle. Electroporation was performed at days 0, 14, and 28. Animals were culled at day 42. (**B**) Coimmunostaining for AQP4 and Myc-DDK in skeletal muscle. Nuclei were counterstained with DAPI. (**C**) H&E staining for skeletal muscle. Images are representative of the longitudinal section of the tibialis anterior muscle from 5 mice per group. Insets are higher-magnification photomicrographs showing immune cell infiltration. (**D**) Percentage area of AQP4 and Myc-DDK colocalization in the muscle. (**E**) Percentage area of necrotic inflammation in the muscle. (**F**) Western blot analysis of protein from skeletal muscle cell lysate separated in native form by BN-PAGE, and in denatured form by SDS-PAGE. Top: BN-PAGE shows the expression of fusion proteins consisting of Myc-DDK and AQP4 OAPs, IMPs, and tetramers in the muscle after electroporation with pAQP4. Bottom: SDS-PAGE shows the expression of a fusion protein (60 kDa) consisting of Myc-DDK and AQP4 M23 monomers in the muscle after electroporation with pAQP4. Data are mean ± SEM; *n* = 5 per group. ***P < 0.001, 1-way ANOVA with post hoc Tukey’s test. Scale bar: 50 μm. Original magnification, ×400 (insets).

The M23 isoform of AQP4 exists in the plasma membrane as homotetrameric and heterotetrameric units, which aggregate to form intramembranous particles (IMPs) and high-order orthogonal arrays of particles (OAPs) (29, 30). To transfect the tibialis anterior muscle of each mouse, we used in vivo electroporation with a plasmid encoding the M23 isoform of mouse AQP4 (pAQP4) and an empty plasmid vector control (pEmpty). The distribution of AQP4 in muscle fibers after electroporation was visualized by immunostaining. The muscle from naive mice showed weak and diffuse distribution of AQP4 immunoreactivity and no Myc-DDK staining. The muscle from the mice that received pEmpty showed weak AQP4 immunoreactivity but strong Myc-DDK staining. Electroporation with pAQP4 increased the number and fluorescent intensity of AQP4- and Myc-DDK–costained muscle fibers with punctate distribution of immunoreactivity suggestive of OAP lattices (*P* *<* 0.001; Figure 1, B and D). Transfection of HEK293 cells with pAQP4 followed by immunostaining confirmed that AQP4 was expressed on the membrane of the transfected cells with an OAP lattice–like structure (Supplemental Figure 1; supplemental material available online with this article; https://doi.org/10.1172/jci.insight.161003DS1). H&E staining revealed prominent necrotic inflammation in the muscle following electroporation as characterized by marked immune cell infiltration (*P* *<* 0.001; Figure 1, C and E).

To characterize AQP4 expression in tibialis anterior muscle after electroporation, we performed Blue native PAGE (BN-PAGE) to separate the native forms of AQP4 tetramers, IMPs and OAPs. Immunoblot analysis revealed that electroporation with pAQP4 profoundly increased the expression of M23 tetramers, IMPs, and OAPs in the muscle compared with naive and pEmpty controls (Figure 1F, top). Next, we resolved denatured proteins from the muscle by SDS-PAGE. Immunoblot analysis revealed that electroporation with pAQP4 robustly increased the expression of a 60 kDa fusion protein, which represents AQP4 monomers together with Myc-DDK, in the muscle compared with naive and pEmpty controls (Figure 1F, bottom). These findings indicate that electroporation with pAQP4 induces the overexpression of AQP4 M23 isoform and triggers inflammation in mouse skeletal muscle.

### AQP4 immunization generates circulating AQP4 autoantibodies.

**Figure 2:**
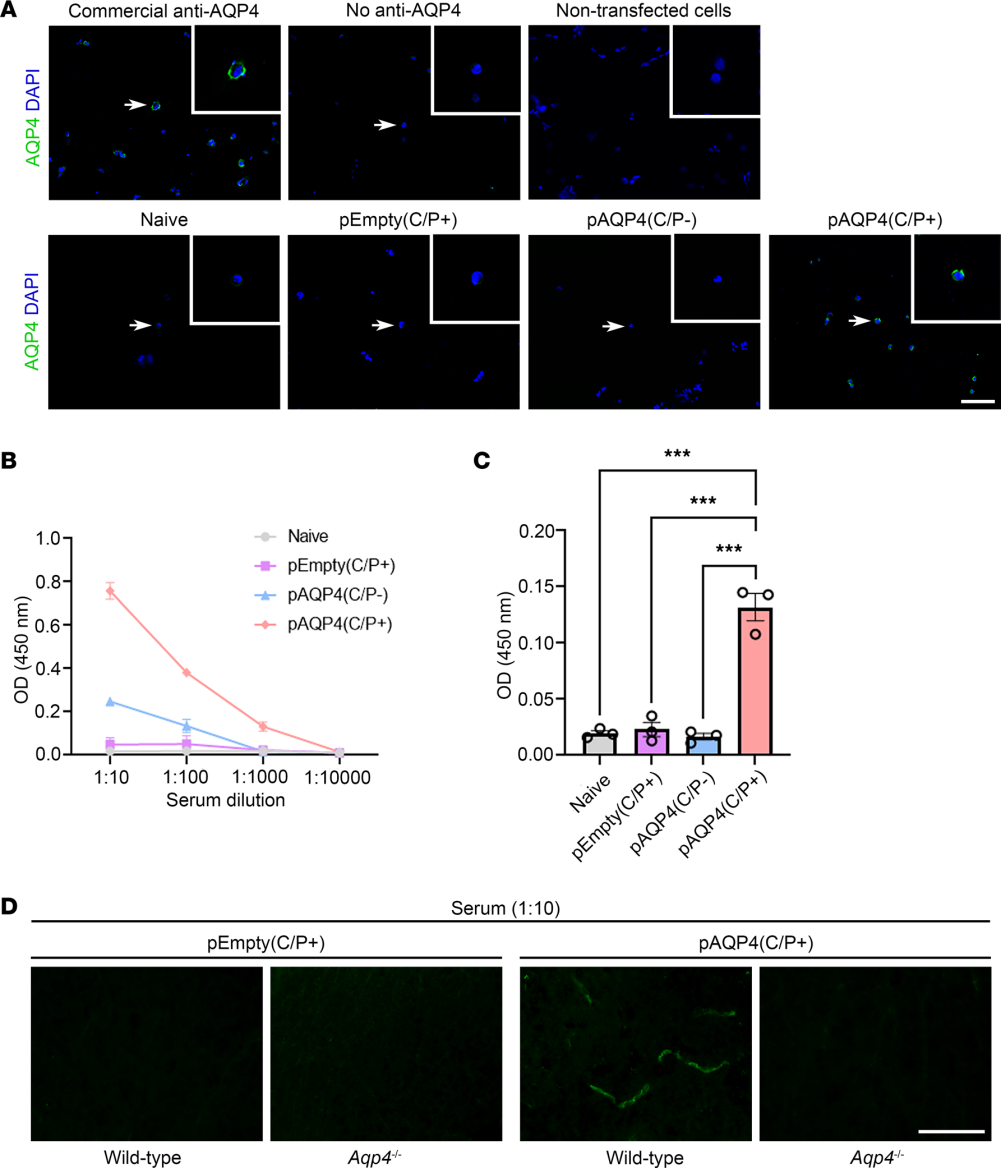
AQP4 immunization generates AQP4 autoantibodies. (**A**) Detection of AQP4 autoantibodies in mouse serum by cell-based indirect immunofluorescence assay. HEK293 cells were transfected with a plasmid encoding the mouse AQP4 M23 isoform. AQP4 autoantibodies in the serum were visualized by fluorescence-conjugated secondary antibody specific for mouse IgG. Top left: Immunostaining with commercial anti-AQP4 antibody revealed a discontinuous pattern of AQP4 staining on the cell membrane (positive control). Top middle and right: No AQP4 immunoreactivity was observed when commercial anti-AQP4 antibody was absent or HEK293 cells were not transfected (negative controls). Bottom: Immunostaining of transfected HEK293 cells using the serum of naive, pEmpty(C/P+), pAQP4(C/P–), and pAQP4(C/P+) mice. Nuclei were counterstained with DAPI. Images are representative of 8 mice per group. Scale bar: 50 μm. Original magnification, ×400 (insets). (**B**) Titer of AQP4 autoantibodies was measured by ELISA using serial dilution of serum from 1:10 to 1:10,000. (**C**) Concentration of AQP4 autoantibodies was determined using serum diluted at 1:1,000. (**D**) Spinal cord sections of WT and *Aqp4*-deficient (*Aqp4^–/–^*) mice were immunostained using the serum of pEmpty(C/P+) and pAQP4(C/P+) mice. Images are representative of 3 mice per group. Data are mean ± SEM; *n* = 3 per group. ***P < 0.001, 1-way ANOVA with post hoc Tukey’s test. Scale bar: 50 μm.

We have shown that cell-based indirect immunofluorescence assay using transfected HEK293 cells expressing human AQP4 on the cell membrane is sensitive and specific for the detection of AQP4-IgG in patients with NMOSD (31). To detect AQP4 autoantibodies in mouse serum after AQP4 immunization, we performed the same assay but used transfected HEK293 cells expressing the mouse AQP4 M23 isoform. Signal was visualized by mouse IgG–specific fluorescent-conjugated secondary antibody. In the positive control using commercial anti-AQP4 antibody to stain the transfected cells, we found positive AQP4 immunoreactivity on the membrane of the cells. In the negative controls, with no commercial anti-AQP4 antibody added and without transfection of the cells, no AQP4 immunoreactivity was observed (Figure 2A, top). Moreover, no AQP4 immunoreactivity was detected when the serum of naive, pEmpty(C/P+), and pAQP4(C/P–) mice was used to stain the cells. However, positive AQP4 immunoreactivity was observed when the serum of pAQP4(C/P+) mice was used (Figure 2A, bottom). These results indicate that the serum of pAQP4(C/P+) mice contains autoantibodies that recognize the M23 isoform of mouse AQP4, including its conformational epitopes.

To determine the titer of AQP4 autoantibodies, the serum of mice in different groups was analyzed using competitive ELISA. No AQP4 autoantibodies were detected in the serum of naive and pEmpty mice. A low titer of AQP4 autoantibodies was found in the serum of pAQP4(C/P–) mice. By contrast, the serum of pAQP4(C/P+) mice displayed a higher titer of AQP4 autoantibodies (Figure 2B). Using serum diluted in 1:1,000, the concentration of AQP4 autoantibodies in pAQP4(C/P+) mice was significantly higher than that in naive, pEmpty(C/P+), and pAQP4(C/P–) mice (*P* < 0.001; Figure 2C). These results support that AQP4 immunization via in vivo electroporation triggers the production of AQP4 autoantibodies in mouse circulation.

AQP4 is predominantly located on the astrocytic foot processes at the BBB (32). Next, we tested whether the serum from AQP4-immunized mice could immunostain the spinal cord sections that were obtained from WT and *Aqp4*-deficient (*Aqp4*^–/–^) mice. There was no detectable immunoreactivity when the serum of pEmpty(C/P+) mice was used to stain the spinal cord sections from WT mice and *Aqp4^–/–^* mice. However, when the serum of pAQP4(C/P+) mice was used, positive staining was found at the blood vessels of the spinal cord from WT mice, but not at that from *Aqp4^–/–^* mice (Figure 2D). These results further confirm that AQP4-immunized mice contain circulating AQP4 autoantibodies.

### AQP4 immunization induces motor impairments.

**Figure 3:**
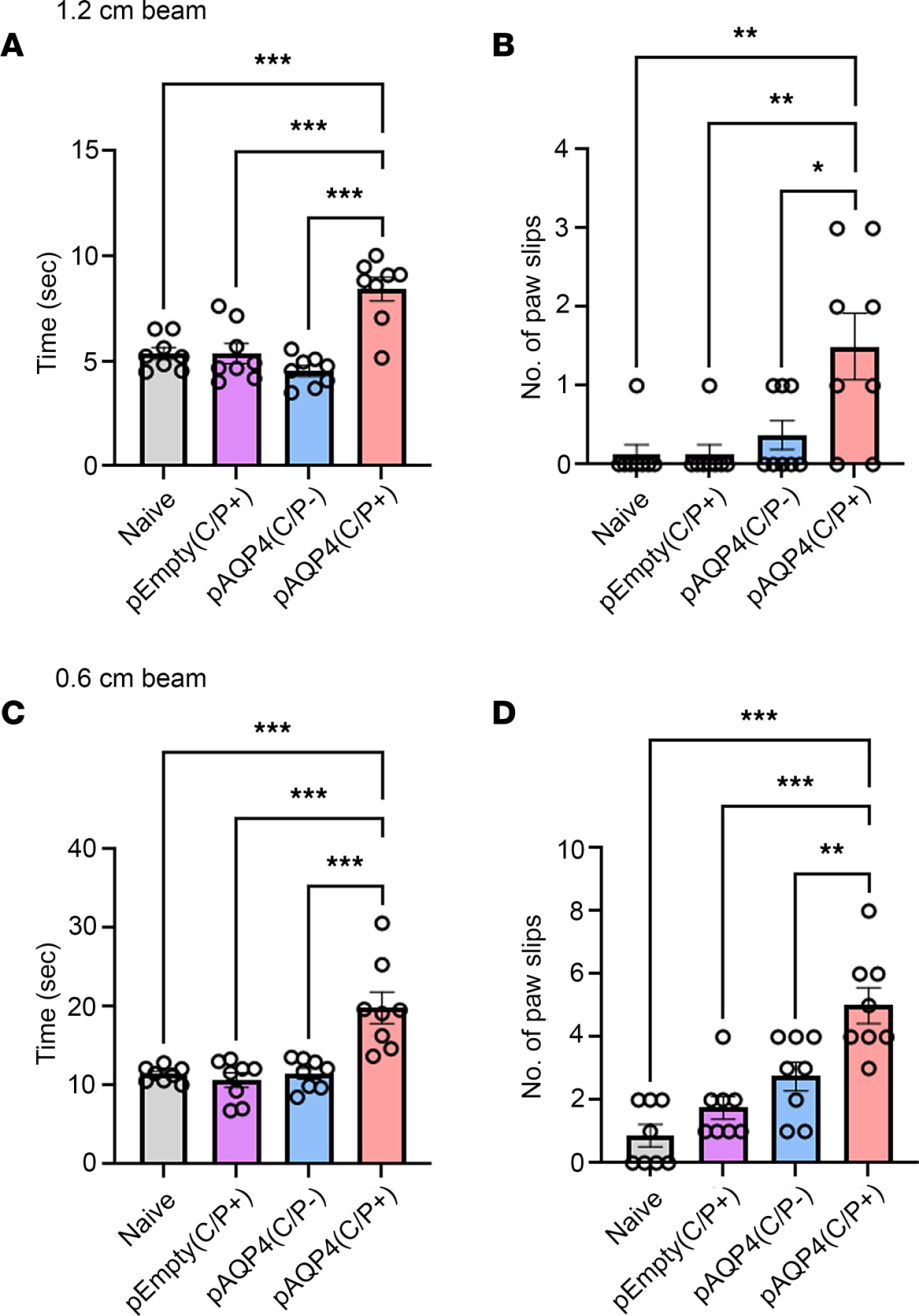
AQP4 immunization induces motor impairments. (**A** and **B**) Beam walking test measuring time taken by a mouse to cross and number of paw slips while crossing a 1.2 × 80 cm (width × length) beam. (**C** and **D**) Time taken to cross and number of paw slips while crossing a 0.6 × 80 cm beam. Data are mean ± SEM; *n* = 8 per group. *P < 0.05, **P < 0.01, ***P < 0.001, 1-way ANOVA with post hoc Tukey’s test.

Clinical signs of encephalomyelitis were assessed by experimental autoimmune encephalomyelitis score. From day 0 to day 42, pAQP4(C/P+) mice did not display motor weakness compared with naive, pEmpty(C/P+), and pAQP4(C/P–) mice (score 0, data not shown). At day 42, we examined whether AQP4 immunization led to motor impairments using a beam walking test. pAQP4(C/P+) mice took a longer time to cross a 1.2 × 80 cm (width × length) beam than naive, pEmpty(C/P+), and pAQP4(C/P–) mice (*P* < 0.001; Figure 3A). During the beam walking test, pAQP4(C/P+) mice slipped more often than control mice (*P* < 0.05; Figure 3B). Similar findings were observed when the test was performed with a narrower 0.6 × 80 cm beam. pAQP4(C/P+) mice took a longer time (*P* < 0.001; Figure 3C) and slipped more often (*P* < 0.01; Figure 3D) than controls. These results indicate that AQP4-immunized mice displayed motor impairments.

### Circulating AQP4 autoantibodies infiltrate the spinal cord.

**Figure 4:**
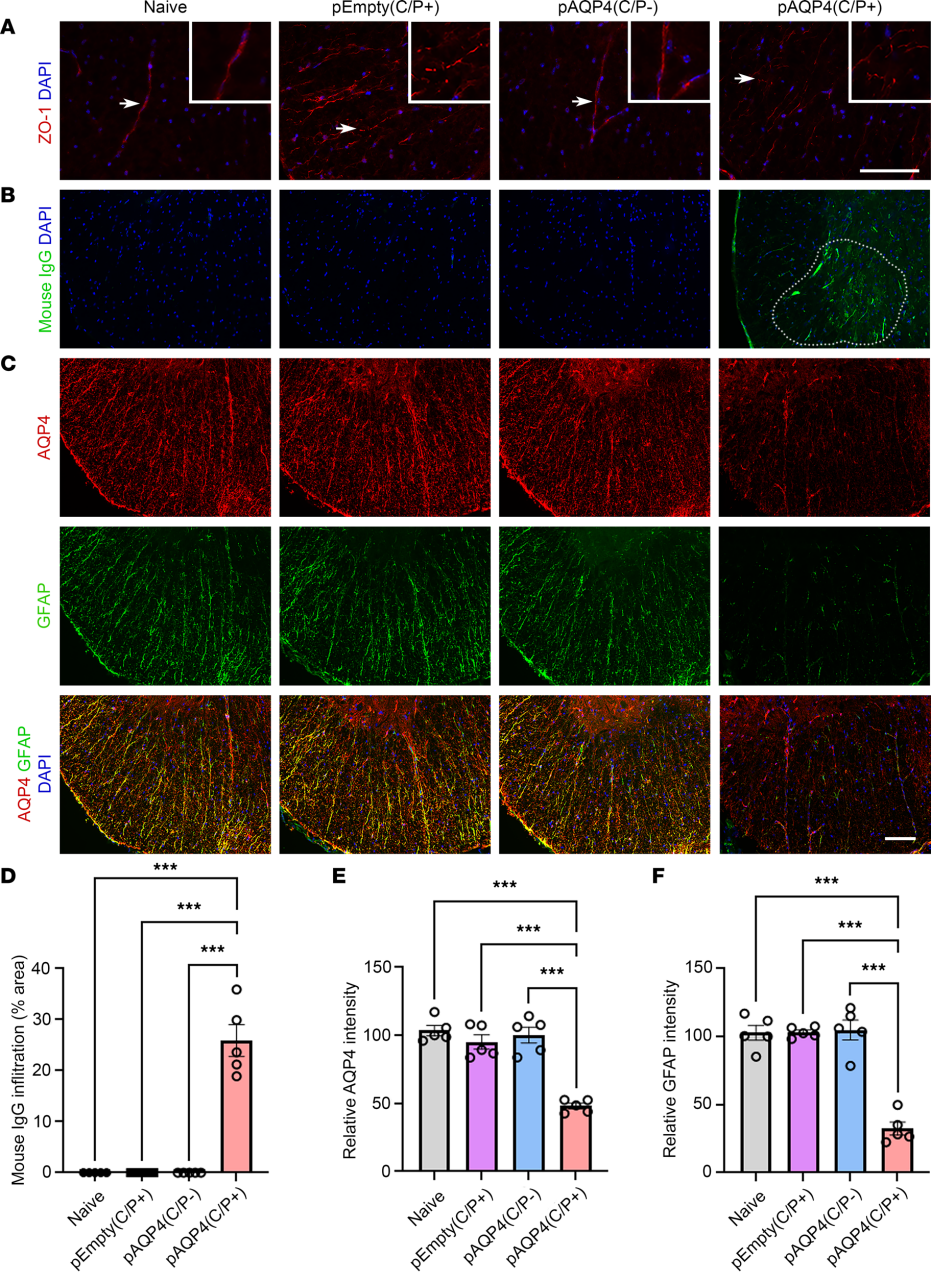
IgG infiltration and astrocytopathy after AQP4 immunization. (**A**) Immunostaining for ZO-1 in the spinal cord of naive, pEmpty(C/P+), pAQP4(C/P–), and pAQP4(C/P+) mice. Insets are higher-magnification photomicrographs showing the pattern of ZO-1 staining in blood vessels. Original magnification, ×400 (insets). (**B**) Immunostaining for mouse IgG in the spinal cord of naive, pEmpty(C/P+), pAQP4(C/P–), and pAQP4(C/P+) mice. Dotted line demarcates the area of mouse IgG immunoreactivity. (**C**) Coimmunostaining for AQP4 and GFAP in the spinal cord of naive, pEmpty(C/P+), pAQP4(C/P–), and pAQP4(C/P+) mice. (**D**) Quantification of mouse IgG infiltration. (**E** and **F**) Quantification of AQP4 and GFAP immunofluorescence intensities. Images are representative photomicrographs showing the ventrolateral white matter of cervical spinal cord cross sections from 5 mice per group. Nuclei were counterstained with DAPI. Data are mean ± SEM; *n* = 5 per group. ***P < 0.001, 1-way ANOVA with post hoc Tukey’s test. Scale bars: 50 μm.

Without passing through the BBB, circulating AQP4-IgGs in the peripheral blood do not cause CNS damage and acute attack in NMOSD (24). To disrupt the BBB in this model, mice were injected with CFA and pertussis toxin (PTx) before electroporation as described previously (27, 33). At day 42, immunostaining for the tight junction protein ZO-1 revealed a continuous staining pattern in the spinal cord blood vessels of naive and pAQP4(C/P–) mice, but a discontinuous pattern in those of pEmpty(C/P+) and pAQP4(C/P+) mice (Figure 4A). These results indicate that CFA and PTx injections can disrupt the BBB.

Next, we investigated whether circulating AQP4 autoantibodies generated by AQP4 immunization infiltrated the spinal cord after BBB disruption. Mouse IgG immunoreactivity was found in the spinal cord parenchyma of pAQP4(C/P+) mice, but not in that of controls (Figure 4, B and D). Coimmunostaining confirmed that these mouse IgGs targeted AQP4 in the spinal cord (Supplemental Figure 2). These findings confirm that circulating AQP4 autoantibodies infiltrate the spinal cord through a disrupted BBB.

### AQP4 immunization induces astrocytopathy.

A hallmark histopathological feature of NMOSD lesions is astrocytopathy characterized by prominent loss of AQP4 and GFAP (34). We assessed whether AQP4 immunization induces astrocytopathy in our model. Coimmunostaining revealed a profound decrease in AQP4 and GFAP levels in the spinal cord of pAQP4(C/P+) mice compared with naive, pEmpty(C/P+), and pAQP4(C/P–) mice (Figure 4C). Merging of the images showed that the loss of AQP4 colocalized with the loss of GFAP immunoreactivity (Figure 4C). Quantification of immunofluorescence intensity confirmed a significant reduction in AQP4 and GFAP levels in pAQP4(C/P+) mice (*P* < 0.001; Figure 4, E and F). Similar results were observed in the optic nerve of mice in different groups (Supplemental Figure 3, A, C, and D). These data suggest that AQP4 autoantibodies generated by AQP4 immunization cause astrocytopathy in mouse CNS.

### AQP4 immunization induces demyelination and axonal loss.

**Figure 5:**
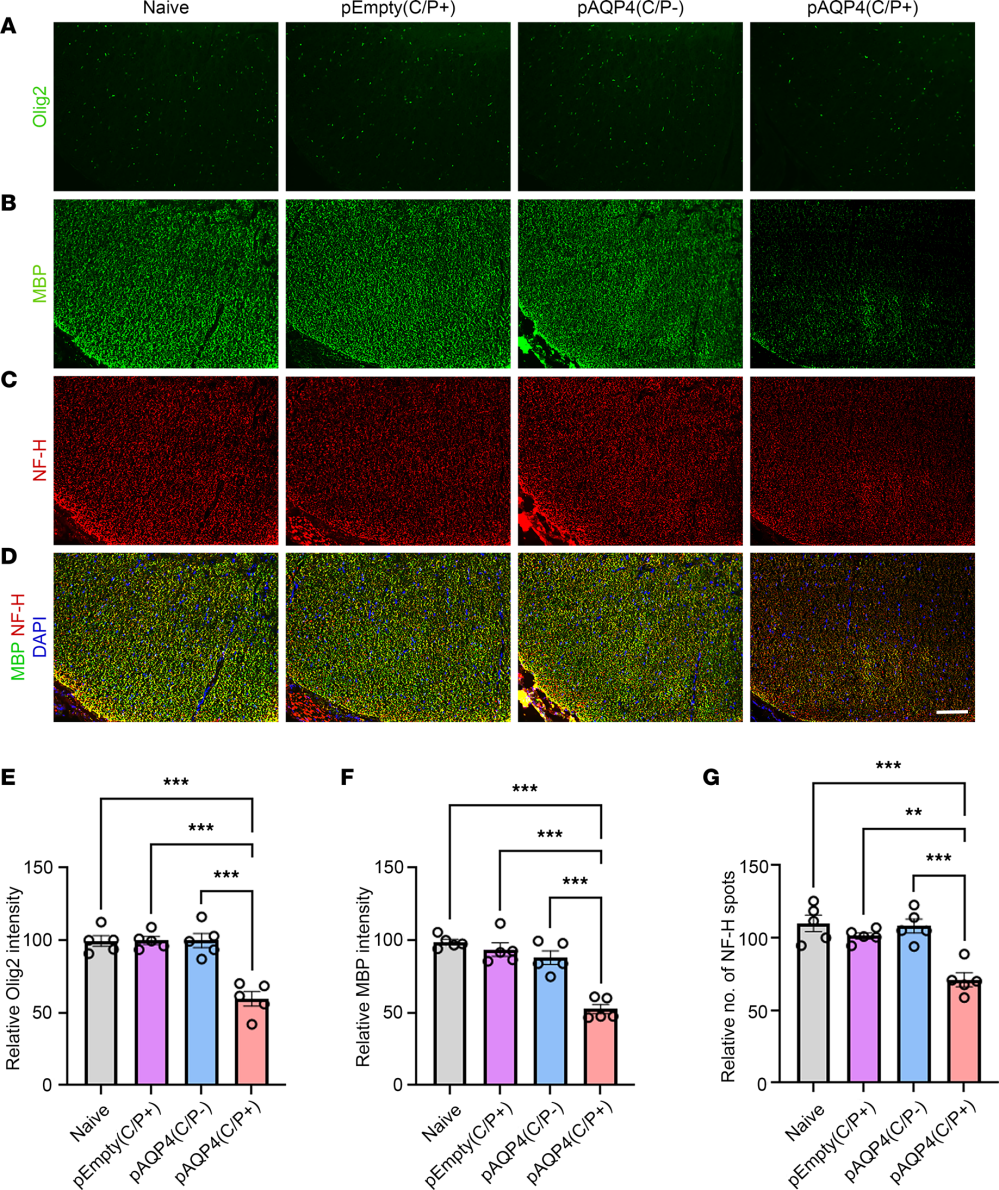
Oligodendrocyte loss, demyelination, and axonal loss after AQP4 immunization. (**A**–**C**) Immunostaining for Olig2 (**A**), MBP (**B**), and NF-H (**C**) in the spinal cord of naive, pEmpty(C/P+), pAQP4(C/P–), and pAQP4(C/P+) mice. (**D**) Merged images of MBP and NF-H coimmunostaining. (**E** and **F**) Quantification of Olig2 and MBP immunofluorescence intensities. (**G**) Quantification of the number of NF-H spots. Images are representative photomicrographs showing the ventrolateral white matter of cervical spinal cord cross sections from 5 mice per group. Nuclei were counterstained with DAPI. Data are mean ± SEM; *n* = 5 per group. **P < 0.01, ***P < 0.001, 1-way ANOVA with post hoc Tukey’s test. Scale bar: 50 μm.

Next, we assessed the effect of AQP4 immunization on demyelination, which is another histopathological feature of NMOSD lesions (34). The spinal cord of pAQP4(C/P+) mice displayed patchy loss of Olig2 (oligodendrocyte marker; Figure 5A) and myelin basic protein (MBP; myelin marker; Figure 5B) immunoreactivities, while this loss was not observed in naive, pEmpty(C/P+), and pAQP4(C/P–) mice. Quantification of immunofluorescence intensity showed significant decrease in Olig2 (*P* < 0.001; Figure 5E) and MBP (*P* < 0.001; Figure 5F) levels in pAQP4(C/P+) mice. Luxol fast blue staining confirmed that demyelination occurred in the spinal cord of pAQP4(C/P+) mice, but not in that of controls (Supplemental Figure 4A).

We further examined axonal loss after AQP4 immunization using neurofilament-heavy (NF-H) as an axonal marker. NF-H–positive spots in the spinal cord of pAQP4(C/P+) mice were less dense than those in controls (Figure 5C). Counting of NF-H spots confirmed significant axonal loss in pAQP4(C/P+) mice (*P* < 0.001; Figure 5G). Coimmunofluorescence of MBP and NF-H revealed an association of demyelination with axonal loss (Figure 5D). These findings were also observed in the optic nerve of mice in different groups (Supplemental Figure 3, B, E, and F). Fluorescent Nissl staining did not show any loss of motoneurons in the ventral horn and interneurons around the central canal in the spinal cord of pAQP4(C/P+) mice (Supplemental Figure 4, B and C). Taken together, these results indicate that AQP4 immunization leads to demyelination and axonal loss.

### AQP4 immunization activates microglia.

**Figure 6:**
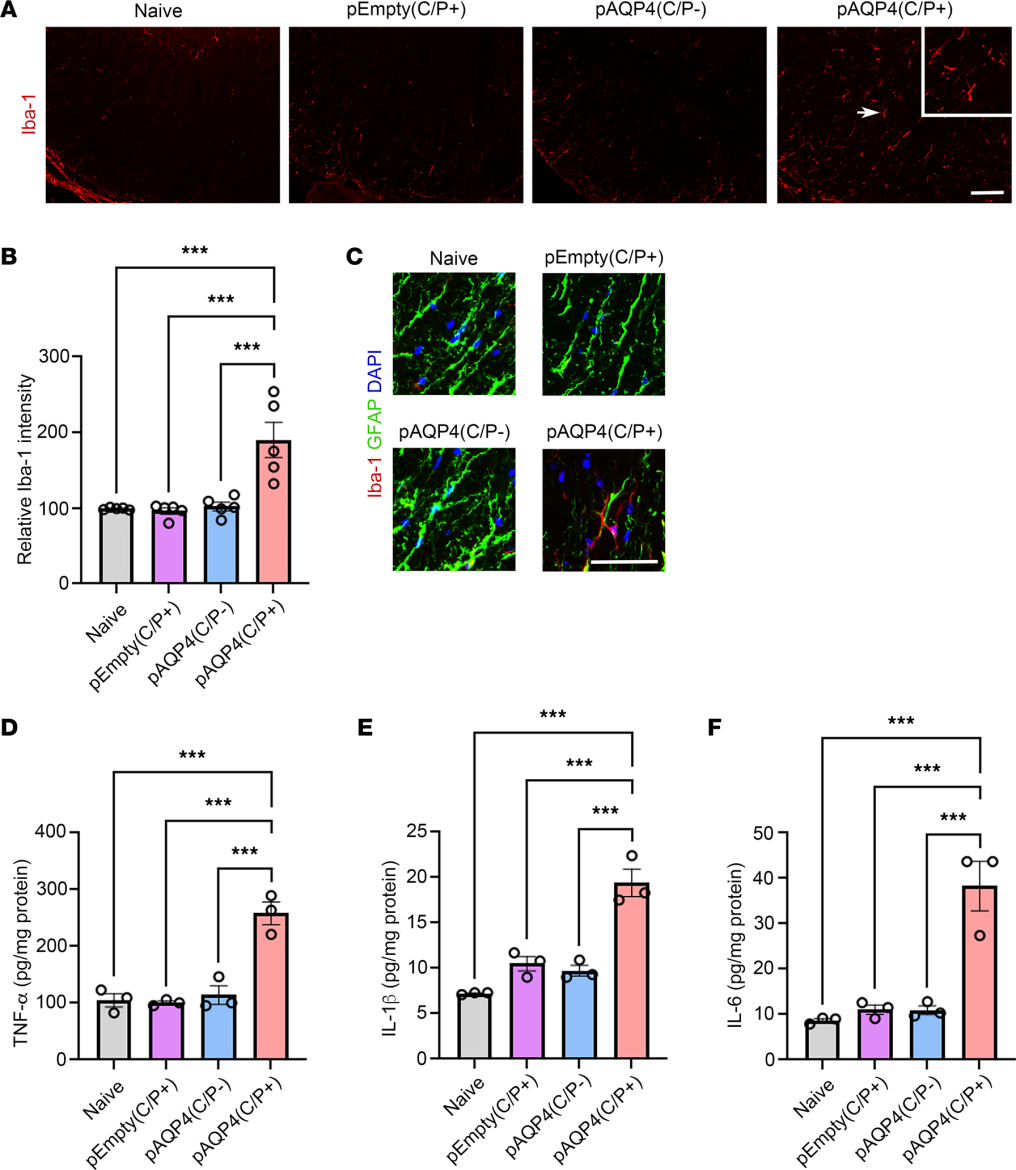
Microglia activation and increase in proinflammatory cytokine levels after AQP4 immunization. (**A**) Immunostaining for Iba-1 in the spinal cord of naive, pEmpty(C/P+), pAQP4(C/P–), and pAQP4(C/P+) mice. Scale bar: 50 μm. Original magnification, ×400 (inset). (**B**) Quantification of Iba-1 immunofluorescence intensity. (**C**) Coimmunostaining for Iba-1 and GFAP. (**D**–**F**) ELISA of TNF-α, IL-1β, and IL-6 levels in the spinal cord homogenate of naive, pEmpty(C/P+), pAQP4(C/P–), and pAQP4(C/P+) mice. Images are representative photomicrographs showing the ventrolateral white matter of cervical spinal cord cross sections from 5 mice per group. Nuclei were counterstained with DAPI. Data are mean ± SEM; *n* = 5 per group (**B**), *n* = 3 per group (**D**–**F**). ***P < 0.001, 1-way ANOVA with post hoc Tukey’s test. Scale bar: 50 μm.

Prominent microglia activation has been found in the lesions of patients with NMOSD and previous experimental models of NMOSD (10, 22, 23, 28, 35). In the present model, we examined whether astrocytopathy, demyelination, and axonal loss coincide with microglia activation. We used Iba-1 as a monocyte marker that labels both brain-derived microglia and infiltrated macrophages. The spinal cord of pAQP4(C/P+) mice displayed a marked increase in Iba-1 immunoreactivity compared with naive, pEmpty(C/P+), and pAQP4(C/P–) mice (Figure 6A). Quantification of immunofluorescence intensity confirmed a significant increase in Iba-1 level in pAQP4(C/P+) mice (*P* < 0.001; Figure 6B). Coimmunostaining of Iba-1 and GFAP revealed that activated microglia were in close proximity to astrocytes (Figure 6C).

Activated microglia release excessive proinflammatory cytokines, including TNF-α, IL-1β, and IL-6, to mediate neuroinflammation (36). To assess the level of these cytokines in our model, ELISA was performed in the spinal cord homogenate of mice in different groups. The level of TNF-α, IL-1β, and IL-6 in pAQP4(C/P+) mice was significantly higher than that in naive, pEmpty(C/P+), and pAQP4(C/P–) mice (*P* < 0.001; Figure 6, D–F). These results suggest a role of microglia activation in the development of NMOSD-like lesions in AQP4-immunized mice.

### Complement activation and immune cell infiltration in AQP4-immunized mice.

Next, we assessed whether AQP4 immunization induces complement complex deposition and immune cell infiltration. C5b-9 (terminal complement complex marker) immunoreactivity was observed in the spinal cord of pAQP4(C/P+) mice, but not in that of naive, pEmpty(C/P+), and pAQP4(C/P–) mice (Supplemental Figure 5A). Scattered Ly6G-immunoreactive (neutrophil marker) and Siglec-F–immunoreactive (eosinophil marker) cells were found in the spinal cord of pAQP4(C/P+) mice, but not in that of controls (Supplemental Figure 5, B and C).

CD68-immunoreactive cells, which represented both brain-derived and infiltrated macrophages, were observed in the spinal cord of pAQP4(C/P+) mice, but not in that of controls (Supplemental Figure 5D). However, sparse F4/80-immunoreactive cells, which represented infiltrated macrophages, were found in the spinal cord of pAQP4(C/P+) mice (Supplemental Figure 5E). These results suggest that only a few circulating macrophages infiltrated the spinal cord parenchyma.

H&E staining confirmed prominent immune cell infiltration in the spinal cord of pAQP4(C/P+) mice (Supplemental Figure 5F). We did not find any immunoreactivity for CD49b (natural killer cells), CD19 (B cells), and CD4 (Th cells) in the spinal cord of mice in all groups (data not shown).

### Aqp4^–/–^ mice do not display NMOSD-like disease activity after AQP4 immunization.

**Figure 7:**
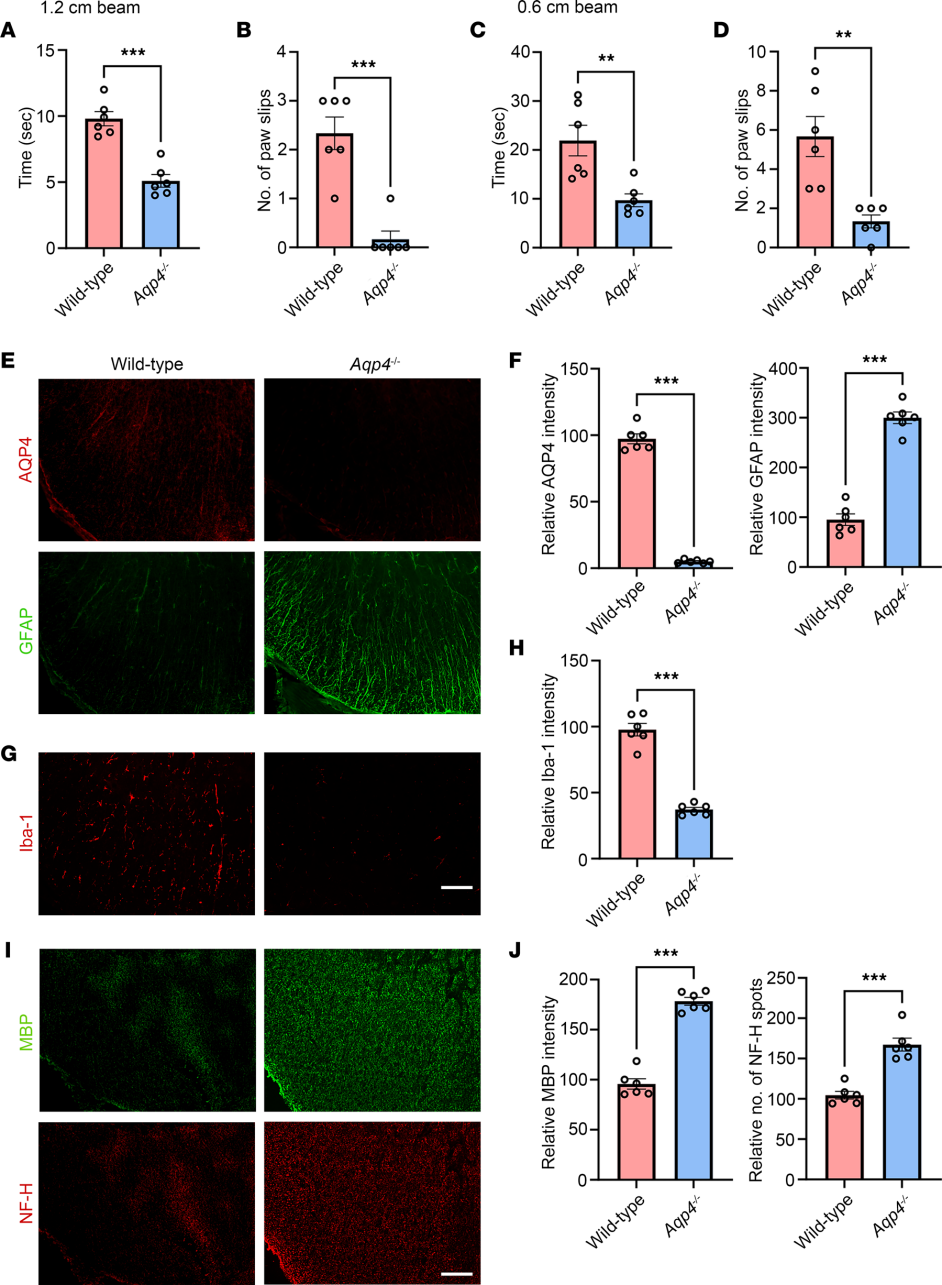
AQP4 immunization does not induce motor impairments and spinal cord pathologies in *Aqp4*-deficient mice. WT or *Aqp4*-deficient (*Aqp4^–/–^*) mice received in vivo electroporation of pAQP4 at the left tibialis anterior muscle. Electroporation was performed at days 0, 14, and 28. Animals were culled at day 42. (**A** and **B**) Beam walking test measuring time taken by a mouse to cross and number of paw slips while crossing a 1.2 × 80 cm (width × length) beam. (**C** and **D**) Time taken to cross and number of paw slips while crossing a 0.6 × 80 cm beam. (**E**) Immunostaining for AQP4 and GFAP in the spinal cord of WT pAQP4(C/P+) and *Aqp4^–/–^* pAQP4(C/P+) mice. (**F**) Quantification of AQP4 and GFAP immunofluorescence intensities. (**G**) Immunostaining for Iba-1 in the spinal cord of WT pAQP4(C/P+) and *Aqp4^–/–^* pAQP4(C/P+) mice. (**H**) Quantification of Iba-1 immunofluorescence intensity. (**I**) Immunostaining for MBP and NF-H in the spinal cord of WT pAQP4(C/P+) and *Aqp4^–/–^* pAQP4(C/P+) mice. (**J**) Quantification of MBP immunofluorescence intensity and number of NF-H spots. Images are representative photomicrographs showing the ventrolateral white matter of cervical spinal cord cross sections from 6 mice per group. Data are mean ± SEM; *n* = 6 per group. **P < 0.01, ***P < 0.001, Student’s 2-tailed *t* test. Scale bars: 50 μm.

To confirm that this experimental disease model is mediated by an autoimmune reaction to AQP4, we performed AQP4 immunization in *Aqp4^–/–^* mice and WT littermate controls. Beam walking test revealed that WT pAQP4(C/P+) mice took a longer time to cross a 1.2 × 80 cm (width × length) beam than *Aqp4^–/–^* pAQP4(C/P+) mice (*P* < 0.001; Figure 7A). WT pAQP4(C/P+) mice slipped more often than *Aqp4^–/–^* pAQP4(C/P+) mice (*P* < 0.001; Figure 7B). Similar findings were observed when the test was performed with a 0.6 × 80 cm beam (Figure 7, C and D). Next, we examined whether *Aqp4^–/–^* mice displayed NMOSD-like pathologies after AQP4 immunization. As expected, the spinal cord of *Aqp4^–/–^* pAQP4(C/P+) mice did not show any AQP4 immunoreactivity. Importantly, there was a significant difference in GFAP immunoreactivity between the spinal cords of *Aqp4^–/–^* pAQP4(C/P+) and WT pAQP4(C/P+) mice (*P* < 0.001; Figure 7, E and F). Moreover, the spinal cord of *Aqp4^–/–^* pAQP4(C/P+) mice displayed profound reduction in Iba-1 immunoreactivity compared with that of WT pAQP4(C/P+) mice (*P* < 0.001; Figure 7, G and H). There was no patchy loss of MBP immunoreactivity and decrease in the number of NF-H spots in *Aqp4^–/–^* pAQP4(C/P+) mice compared with WT pAQP4(C/P+) mice (*P* < 0.001; Figure 7, I and J). These results indicate that the motor impairments and NMOSD-like pathologies observed in this model are driven by an autoimmune response against AQP4, hence they are not observed in immunized *Aqp4^–/–^* mice.

### AQP4 immunization induces the expansion of splenic Tfh, Th1, Th17, memory B, and plasma cells.

Tfh cells drive GC responses with B cell proliferation, differentiation, isotype switching, somatic hypermutation, and affinity maturation (13). In patients with NMOSD, the frequency of circulating Tfh cells has been found to be correlated with the disease activity (15–18). Consistently, we did not find any difference in the circulating Tfh cell frequency between healthy controls and patients with NMOSD in remission receiving immunosuppressive therapy (Supplemental Table 1 and Supplemental Figure 6). In the present animal model, we observed increases in the frequency of Tfh cells (CD4^+^CXCR5^+^PD-1^+^, *P* < 0.001; Supplemental Figure 7, A and D), Th1 cells (CD4^+^IFN-γ^+^, *P <* 0.001; Supplemental Figure 7, B and E), Th17 cells (CD4^+^IL-17A^+^, *P <* 0.001; Supplemental Figure 7, C and F), memory B cells (CD19^+^CD80^+^, *P <* 0.05; Supplemental Figure 8, A and C), and plasma cells (CD19^–^CD138^+^TACI^+^, *P* < 0.05; Supplemental Figure 8, B and D) in the spleen of pAQP4(C/P+) mice compared with naive, pEmpty(C/P+), and pAQP4(C/P–) mice.

### Anti–ICOS-L antibody ameliorates disease activity in AQP4-immunized mice.

**Figure 8:**
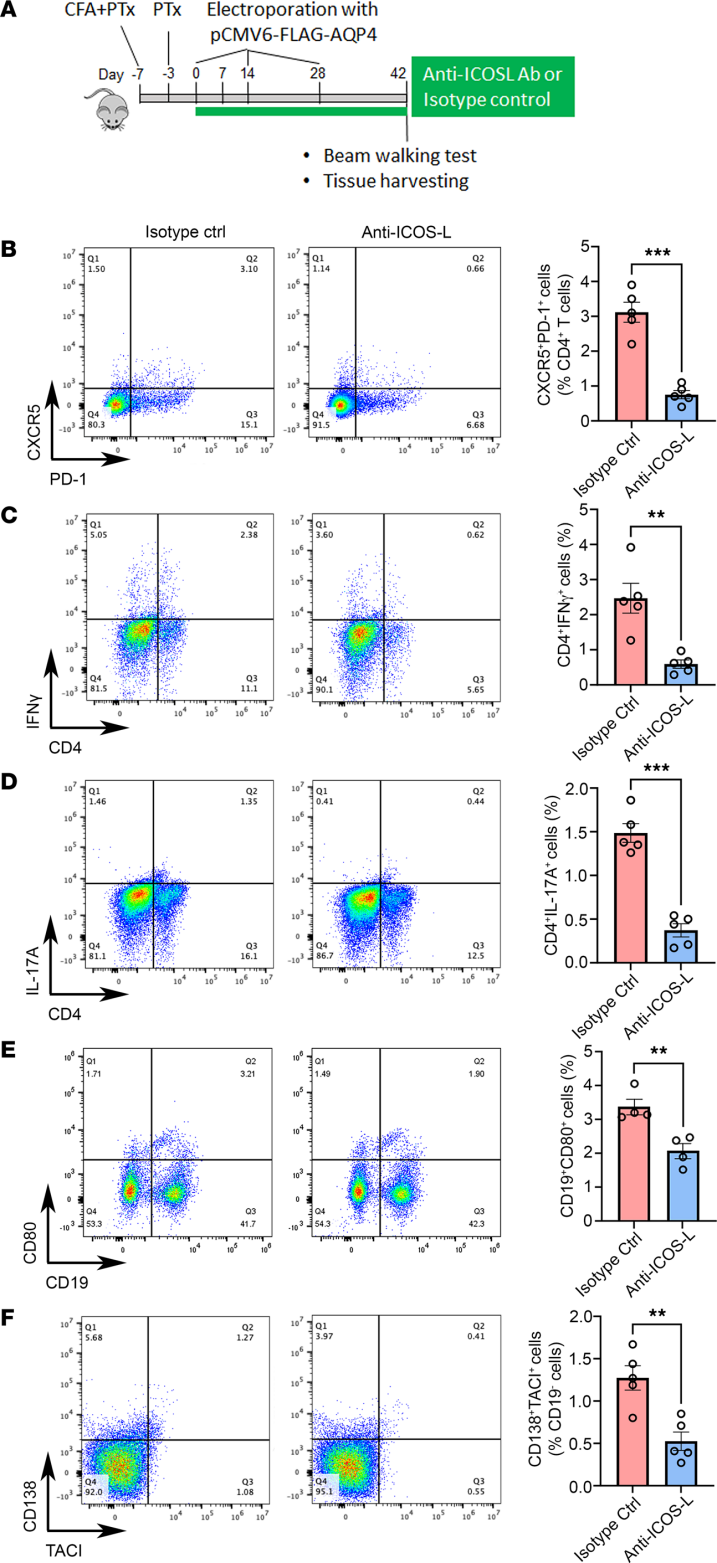
ICOS-L blockade depletes Tfh cells and suppresses Th1, Th17, memory B, and plasma cell responses. (**A**) Experimental design. Beginning at day 0, pAQP4(C/P+) mice were given 150 μg anti–ICOS-L or isotype control antibody (i.p.) 3 times a week. Animals were culled at day 42. (**B**) Representative flow cytometry plots of splenic CXCR5^+^PD-1^+^ Tfh cells following isotype control or anti–ICOS-L treatment, pre-gated on CD4^+^ T cells. (**C**) Representative flow cytometry plots of splenic CD4^+^IFN-γ^+^ Th1 cells following isotype control or anti–ICOS-L treatment. (**D**) Representative flow cytometry plots of splenic CD4^+^IL-17A^+^ Th17 cells following isotype control or anti–ICOS-L treatment. (**E**) Representative flow cytometry plots of splenic CD19^+^CD80^+^ memory B cells following isotype control or anti–ICOS-L treatment. (**F**) Representative flow cytometry plots of splenic CD138^+^TACI^+^ plasma cells following isotype control or anti–ICOS-L treatment, pre-gated on CD19^–^ cells. Flow data are quantified for all groups. Data are mean ± SEM; *n* = 4–5 per group. **P < 0.01, ***P < 0.001, Student’s 2-tailed *t* test.

Tfh cells require a sustained ICOS/ICOS-L signaling to maintain their phenotype (37, 38). Abrogation of the signaling with antibodies targeting ICOS or ICOS-L has been shown to deplete Tfh cells and suppress GC responses (37, 38). To examine whether Tfh cells influence B cell responses, we depleted Tfh cells in pAQP4(C/P+) mice using anti–ICOS-L antibody (Figure 8A). We found that anti–ICOS-L antibody–treated pAQP4(C/P+) mice had fewer splenic Tfh cells than isotype control antibody–treated pAQP4(C/P+) mice (CD4^+^CXCR5^+^PD-1^+^, *P* < 0.001; Figure 8B). Moreover, the frequency of splenic Th1 cells (CD4^+^IFN-γ^+^, *P* < 0.01; Figure 8C), Th17 cells (CD4^+^IL-17A^+^, *P* < 0.001; Figure 8D), memory B cells (CD19^+^CD80^+^, *P* < 0.01; Figure 8E), and plasma cells (CD19^–^CD138^+^TACI^+^, *P* < 0.01; Figure 8F) in anti–ICOS-L antibody–treated pAQP4(C/P+) mice was significantly lower than that in controls.

**Figure 9:**
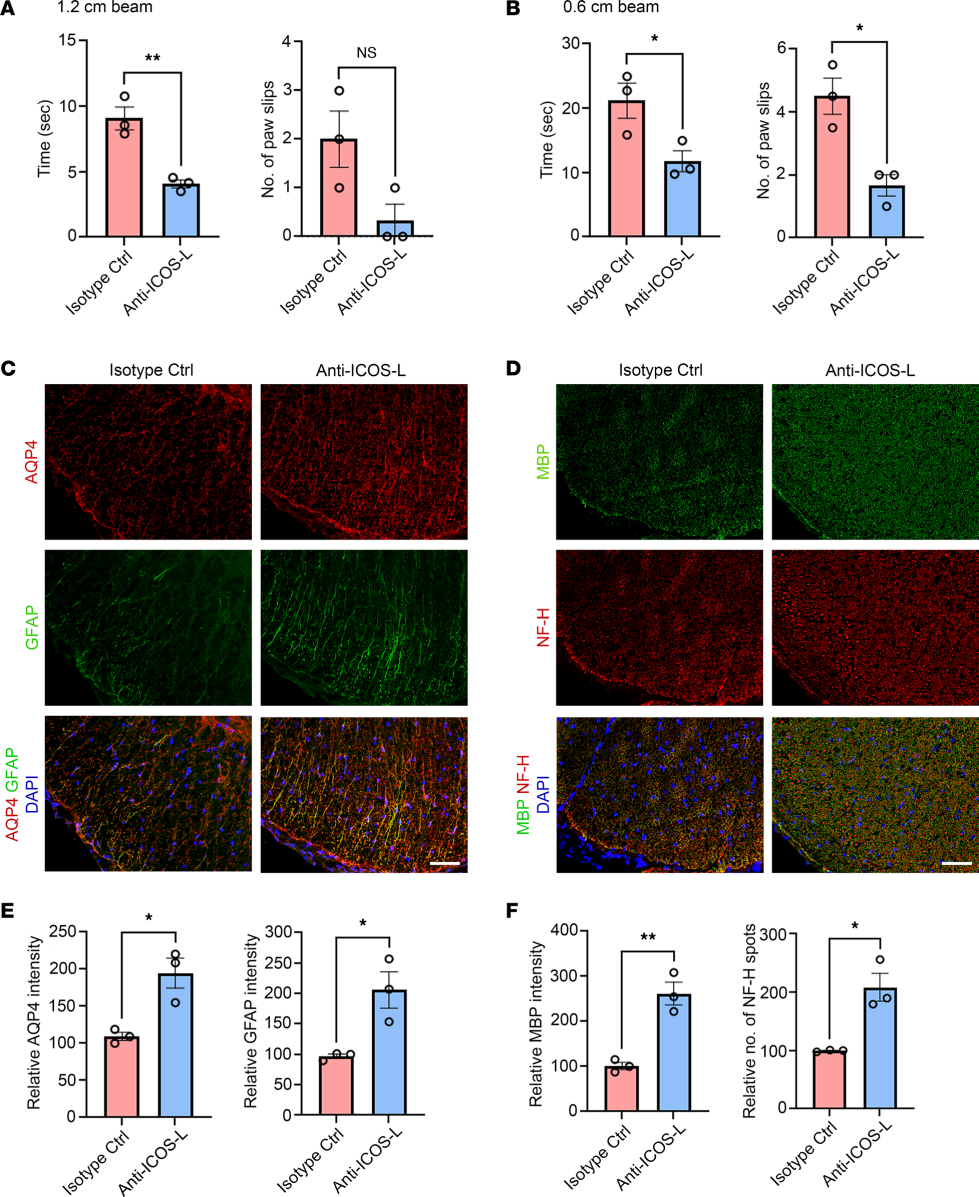
Tfh cell depletion ameliorates NMOSD disease activity. Beginning at day 0, pAQP4(C/P+) mice were given 150 μg anti–ICOS-L or isotype control antibody (i.p.) 3 times a week. Animals were culled at day 42. (**A** and **B**) Beam walking test measuring time taken by a mouse to cross and number of paw slips while crossing a 1.2 × 80 cm beam (**A**) and a 0.6 × 80 cm beam (**B**). (**C**) Coimmunostaining for AQP4 and GFAP in the spinal cord of pAQP4(C/P+) mice following isotype control or anti–ICOS-L treatment. (**D**) Coimmunostaining for MBP and NF-H in the spinal cord of pAQP4(C/P+) mice following isotype control or anti–ICOS-L treatment. (**E**) Quantification of AQP4 and GFAP immunofluorescence intensities. (**F**) Quantification of MBP immunofluorescence intensity and number of NF-H spots. Images are representative photomicrographs showing the ventrolateral white matter of cervical spinal cord cross sections from 3 mice per group. Nuclei were counterstained with DAPI. Data are mean ± SEM; *n* = 3 per group. *P < 0.05, **P < 0.01, Student’s 2-tailed *t* test. Scale bars: 50 μm.

Next, we investigated whether anti–ICOS-L antibody treatment ameliorates NMOSD-like disease activity in AQP4-immunized mice. Beam walking test revealed that anti–ICOS-L antibody–treated pAQP4(C/P+) mice took a shorter time to cross the 1.2 × 80 cm and 0.6 × 80 cm beams than isotype control antibody–treated pAQP4(C/P+) mice (*P* < 0.05; Figure 9, A and B). During the walking test on the 0.6 × 80 cm beam, anti–ICOS-L antibody–treated mice slipped less often than control (*P* < 0.05; Figure 9B). Consistent with improved motor functions, there was less AQP4 and GFAP loss in the spinal cord of anti–ICOS-L antibody–treated pAQP4(C/P+) mice compared with controls (Figure 9C). Quantification of immunofluorescence intensity confirmed that these reductions were significant (*P* < 0.05; Figure 9E). Furthermore, anti–ICOS-L antibody treatment prevented loss of MBP immunoreactivity and NF-H spots in the spinal cord of pAQP4(C/P+) mice compared with controls (Figure 9D). Quantification of MBP immunofluorescence intensity and number of NF-H spots confirmed that these observations were significant (MBP, *P* < 0.01; NF-H, *P* < 0.05; Figure 9F). Taken together, these results suggest that Tfh cell depletion via the abrogation of ICOS/ICOS-L signaling prevents AQP4 immunization–induced motor impairments and NMOSD-like pathologies.

## Discussion

In this study, we demonstrated that AQP4 immunization via in vivo electroporation triggered an autoimmune response that produced autoantibodies against AQP4 in mice. These autoantibodies infiltrated the spinal cord and bound to the AQP4 on astrocytes. AQP4-immunized mice displayed motor impairments and NMOSD-like pathologies including astrocytopathy, demyelination, axonal loss, and microglia activation. These motor impairments and pathologies were not observed in *Aqp4*-deficient mice after AQP4 immunization. Using this model, we demonstrated that the depletion of Tfh cells by abrogation of ICOS/ICOS-L signaling ameliorates NMOSD-like disease activity. Our study provides a novel mouse model of NMOSD with AQP4 autoimmunity in which Tfh cells contribute to its pathophysiology. This model can be used in future studies to elucidate the autoimmune mechanisms underlying the production of AQP4 autoantibodies and to evaluate novel immune cell–targeted therapeutic interventions for NMOSD.

Electroporation-mediated immunization triggers both humoral and cellular immune responses, resulting in the generation of antibodies against the native conformation of a protein of interest in vivo (39, 40). Skeletal muscle is a suitable site for electroporation because it is composed of a large volume of easily accessible postmitotic cells, which allow long-term and stable expression of the transfected gene (41). Square-wave electric pulses enhance the transfection efficiency and expression level of a target protein by folds of magnitude (40, 42). In addition, electric pulses cause damage and inflammation in muscle, resulting in recruitment of immune cells to the immunization site and the development of immunological memory (39). Pretreatment of skeletal muscle with cardiotoxin before electroporation accelerates antibody production through the recruitment of antigen-presenting cells (40). In pAQP4(C/P–) mice, we detected a low titer of AQP4 antibodies by ELISA, but not by cell-based assay. These mice might contain AQP4 autoantibodies targeting linear epitopes of mouse AQP4 at a low titer. On the contrary, in pAQP4(C/P+) mice, we detected autoantibodies that bound to mouse AQP4 by both ELISA and cell-based assay. These findings suggest that AQP4 immunization induces an AQP4-specific autoimmune response. It generates autoantibodies targeting linear and conformational epitopes of AQP4. In the present work, we sought to use electroporation-mediated immunization to trigger the production of autoantibodies against self-AQP4 in mice. Therefore, we used a plasmid that encodes the mouse AQP4. It is possible that immunization using a plasmid encoding AQP4 of other species could produce a stronger immune response and a higher titer of AQP4 antibodies than using the mouse AQP4 plasmid. Previous studies found that immunization with recombinant AQP4 protein or peptides could not induce motor impairments and CNS pathologies in mice (1, 43). NMOSD-like lesions were achieved by coinjection of AQP4-IgG and human complement into the brain and by systemic injection of AQP4-IgG into rodents with disrupted BBB (20, 21, 44, 45). The discrepancy in the findings between these studies and ours suggests that electroporation-mediated immunization is a more efficient method to drive pathogenic AQP4 autoimmunity in rodents.

We found that AQP4-immunized mice displayed signs of motor impairments. This observation is consistent with those from previous murine models, in which AQP4-IgGs from patients with NMOSD were delivered by intracerebral injection (21), intrathecal injection (23), intraventricular infusion (24), or intraperitoneal injection (27). This suggests that the AQP4 autoantibodies produced in our model can lead to clinical motor impairments reminiscent of human AQP4-IgG.

A pathological hallmark of NMOSD is astrocytopathy in the spinal cord and optic nerves, characterized by AQP4 and GFAP losses (1). Various experimental animal models have attempted to recapitulate astrocytopathy in human NMOSD by passive transfer of human AQP4-IgG or AQP4-sensitized T cells to rodents (46). Findings from these models suggest that the binding of AQP4-IgG to astrocytic AQP4 activates the classical complement system, leading to astrocytic loss that is mediated by complement-dependent cytotoxicity (5, 21, 22, 47–49). Interestingly, the presence of serum complement inhibitor CD59 has been suggested to limit the classical complement activity in mouse (50, 51). On the contrary, another study showed that CD59 is broadly expressed in peripheral organs of mice, while its expression is restricted in the brain (52). Following AQP4 immunization, we observed that AQP4 and GFAP losses coincided with C5b-9 deposition. The binding of AQP4 autoantibodies on astrocytic membrane may aggregate the AQP4 M23 isoform to produce OAPs that activate complements (53, 54). Further studies should investigate the role of intrinsic complement inhibitors in lesion development and disease progression in our model. Another mechanism of astrocytopathy in NMOSD has been suggested to involve antibody-dependent cell-mediated cytotoxicity (ADCC). This is triggered by the binding of Fcγ receptors on myeloid cells to the Fc portion of astrocyte-bound AQP4 autoantibodies, leading to the release of cytotoxic compounds and the phagocytosis of astrocytes (55–58). We recently reported that passive transfer of AQP4-IgG from patients with NMOSD to mice induced profound astrocytopathy that was likely mediated by ADCC (27). Here, we observed prominent microglia activation together with neutrophil and eosinophil infiltration in the spinal cord of AQP4-immunized mice. Moreover, activated microglia were in close proximity to astrocytes. Consistent with previous findings, our data further support existing evidence that the astrocytopathy in NMOSD is partly mediated by ADCC.

We found that AQP4-immunized mice displayed prominent oligodendrocyte loss, demyelination, and axonal loss. Oligodendrocytes and neurons do not express AQP4; therefore they are not directly damaged by AQP4-IgG (21). Recent studies have highlighted a complement bystander mechanism that mediates secondary oligodendrocyte and neuronal damage in NMOSD. It involves a local diffusion of activated complements following the binding of AQP4-IgG to astrocytes, resulting in a deposition of membrane attack complex on neighboring oligodendrocytes and neurons (59, 60). Moreover, the secondary damage has been suggested to involve a complement-independent ADCC bystander mechanism (58). This mechanism is mediated by activated microglia and myeloid cells that undergo phagocytosis and produce proinflammatory cytokines and oxidative metabolites (58, 61–63). Most recently, the evolving NMOSD pathophysiology induced by AQP4-IgG infusion in mice has been shown to be dependent on the interaction between astrocytes and microglia that involves a microglial C3a receptor signaling (64). In the present model, oligodendrocyte loss, demyelination, and axonal loss coincided with microglia activation, increased proinflammatory cytokine levels, terminal complement complex deposition, and granulocyte infiltration. These lesion features are consistent with those from previous studies. Taken together, these findings indicate that the binding of AQP4-IgG to astrocytes triggers secondary myelin and neuronal damage that is mediated by complex local inflammatory responses. Our model provides a perspective for further studies on the pathophysiology involved following the binding of AQP4-IgG to astrocytic AQP4.

Our study suggests that Tfh cells contribute to the pathophysiology of NMOSD-like disease in a mouse model with AQP4 autoimmunity induced by AQP4 immunization. Abrogating ICOS/ICOS-L signaling suppressed Tfh cell expansion, reduced Th1, Th17, memory B, and plasma cell responses, and ameliorated motor impairments and NMOSD-like pathologies in AQP4-immunized mice. No significant difference was found in the frequency of circulating Tfh cells between healthy controls and patients with NMOSD in remission receiving immunosuppressive therapy. These findings are consistent with recent clinical studies of circulating Tfh cells in patients with NMOSD. The frequency of circulating Tfh cells during relapse was higher than that during remission, and decrease in the proportion of Tfh cells by methylprednisolone treatment was associated with relapse prevention in patients with NMOSD (15). Circulating Tfh cell and B cell frequencies also closely correlated with the disease activity of NMOSD, and depletion of IL-6–producing B cells inhibited circulating Tfh cell expansion (16, 17). Untreated patients with NMOSD displayed a Tfh polarization toward excessive B helper Tfh subsets, which was restored by B cell depletion therapy (18). These findings support the hypothesis that dysregulated Tfh cells favor B cell differentiation and AQP4-IgG production. To our knowledge, this present model is the first animal model with AQP4 autoimmunity that mimics T cell responses in NMOSD. In experimental autoimmune encephalomyelitis (EAE), a widely used animal model of multiple sclerosis, Tfh cell frequency in the CNS was found to be increased during the peak of the disease (65). The number of infiltrating Tfh cells was correlated with that of infiltrating B cells; and adoptive transfer of Tfh cells increased severity and delayed remission (66). Blocking the infiltration of Tfh cells using anti-CXCL13 antibody alleviated the disease (67). These findings indicate a role of Tfh cells in the pathogenesis of autoimmune neuroinflammation. Future studies on T and B cell interactions using the present model may provide a better understanding of the autoimmune mechanisms leading to AQP4-IgG production. Moreover, future experiments examining the effects of interventions known to suppress NMOSD can add validity to the present model and to the targeting of Tfh cells. Indeed, we plan to examine whether B cell suppression alleviates disease and the associated shift in B and T cell phenotypic profiles using this model in a future study.

A limitation of this study is that we examined the production of AQP4 autoantibodies and NMOSD-like pathologies following AQP4 immunization at one time point only. Further studies are needed to investigate whether AQP4 immunization generates Tfh cells that precede GC expansion. It would also be important to examine the long-term effects of AQP4 immunization on NMOSD-like disease activity in this model. Additionally, Th1, Th17, and T follicular regulatory cells express ICOS, although its expression is highest on GC Tfh cells (14). We cannot exclude the possibility that the effect of anti–ICOS-L antibody observed here was partly influenced by other T cell subsets.

In summary, we demonstrate that AQP4 immunization via electroporation triggers AQP4 autoantibody production that is associated with motor impairments, NMOSD-like pathologies, and increase in splenic Tfh, Th1, Th17, memory B, and plasma cells in mice. Moreover, our results suggest that modulating ICOS/ICOS-L signaling affects Tfh cells, which contribute to the pathophysiology in this novel NMOSD model. Future studies on the precise role of T and B cell subsets in AQP4 autoantibody production and disease severity can extrapolate this model to further understanding of NMOSD pathogenesis. This model may also be useful for evaluating novel therapies for the disease.

## Methods

### Patients with NMOSD

Samples were obtained from 4 healthy controls (HCs) and 6 patients with NMOSD enrolled at the Department of Medicine, Queen Mary Hospital, Hong Kong, from June to November 2022. For patients with NMOSD, inclusion criteria were age ≥18 years and a diagnosis of AQP4-IgG–seropositive NMOSD according to the 2015 International Panel for NMO Diagnosis consensus criteria (68), regardless of the disease course and disease-modifying treatment. Exclusion criteria were another immune disorder, infection, or cancer. AQP4-IgG serostatus was assessed using cell-based indirect immunofluorescence assay in our hospital as reported previously (31). Age, sex, disease duration, current treatment, and relapse/remission status were recorded. Degree of disability was assessed by neurological examination with Expanded Disability Status Scale score. None of the HCs had a history of disease or infection, and none had received any treatment during the previous 2 months. Written informed consent was obtained from all subjects.

### Animals

Female WT C57BL/6N mice (The Jackson Laboratory) of age 6–8 weeks were housed in our animal facilities at the Centre for Comparative Medicine Research, The University of Hong Kong. *Aqp4^–/–^* mice were obtained from Cyagen and were housed under specific pathogen–free conditions. Animals were kept in groups of 4–5 per cage under a 12-hour dark/12-hour light cycle and provided with free access to water and chow.

### Plasmids

Plasmids encoding the mouse AQP4 M23 isoform (pAQP4) and empty vector control (pEmpty) were obtained from OriGene. The pAQP4 plasmid contained mouse AQP4 cDNA inserted into Myc-DDK–tagged expression vector with cytomegalovirus promoter. Plasmids were transformed and amplified in *E*. *coli* competent cells (Clontech). The integrity of the constructs produced was confirmed by DNA sequencing analysis. Plasmid DNA for in vivo electroporation was prepared by Plasmid Purification Maxi Kit (Qiagen) according to the manufacturer’s instructions. The quantity and quality of DNA were assessed by optical density at 260 and 280 nm.

### AQP4 immunization via electroporation

The procedure of animal experiments is summarized in Figure 1A. AQP4 immunization via in vivo electroporation was performed as described previously with modifications (40). At 7 days before the first electroporation, under anesthesia with ketamine (100 mg/kg) and xylazine (10 mg/kg), mice were injected with 10 μM in 50 μL of cardiotoxin (Latoxan) at the right tibialis anterior muscle. Then they received 4 subcutaneous injections of 50 μL complete Freund’s adjuvant (CFA; BD Biosciences) containing 50 μg heat-killed H37Ra *Mycobacterium tuberculosis* (Difco) at 4 sites on the hind flank, 2 at shoulder and 2 at lower back. In addition, mice received intraperitoneal injections of pertussis toxin (PTx; 200 ng in 200 μL PBS; List Biological Laboratories) at 7 and 3 days before the first electroporation.

At day 0, under anesthesia, mice received an intramuscular injection of either pEmpty control or pAQP4 (50 μg DNA in 35 μL PBS) into the right tibialis anterior muscle using a Hamilton syringe (Postnova Analytics). Immediately after DNA injection, square-wave electric pulses (8 pulses of 100 V/cm, duration 20 milliseconds, and frequency 1.0 Hz) generated by an electroporator (ECM830, BTX) were delivered to the muscle using a pair of caliper electrodes (BTX). The electroporation procedure was repeated at days 14 and 28.

Naive mice, without receiving any treatments, served as normal control. Mice that received pEmpty electroporation and were treated with CFA and PTx were termed pEmpty(C/P+); mice that received pAQP4 but without CFA and PTx were termed pAQP4(C/P–); and mice that received both pAQP4 electroporation and CFA and PTx treatment were termed pAQP4(C/P+). Each group contained 8 mice.

### Treatment with anti–ICOS-L antibody

Tfh cells were depleted using a blockade of ICOS/ICOS-L signaling as described previously (38). Beginning at day 0, AQP4-immunized mice were given 150 μg in 200 μL PBS of anti–ICOS-L (clone HK5.3, Bio X Cell) or isotype control (clone 2A3, Bio X Cell) antibody via intraperitoneal injection every other day for 42 days (Figure 8A).

### Motor assessments

All motor assessments were assessed by a researcher blinded to the experimental conditions. Motor weakness of mice was assessed by EAE score as described previously (27, 33). From day 0 to day 42, mice were weighted and examined daily on a 6-grade scale: 0, no clinical signs; 1, weight loss, limp tip of tail; 2, limp tail, mild paraparesis; 3, moderate paraparesis, ataxia; 4, tetraparesis; 5, moribund. Mice were culled if they had weight loss exceeding 20%–30% of initial body weight or developed severe weakness (score 4–5).

At day 42, motor impairments of mice were detected by beam walking test (69). This test examined the animal’s ability to keep upright and walk across an elevated narrow beam to a platform. The apparatus consisted of two 80-cm-long beams with a width of 1.2 and 0.6 cm, resting 50 cm above a table top on 2 stands. The time for the mouse to cross each beam and the number of paw slips during the process were recorded.

### Serum collection and tissue harvesting

After beam walking test, mice were euthanized with an excessive dose of ketamine and xylazine. Blood samples were harvested from the facial vein of the mice with animal lancets (Medipoint). Sera were obtained by collection of the supernatants after centrifuging of the clotted blood samples at 3,100*g* for 15 minutes at 4°C. Tibialis anterior muscles and cervical spinal cords were freshly dissected out, frozen in liquid nitrogen, and stored at –80°C. Some animals were perfused transcardially with ice-cold PBS, followed by 4% paraformaldehyde. Tibialis anterior muscles, cervical spinal cords, and optic nerves were harvested, postfixed with 4% paraformaldehyde, cryoprotected with 30% sucrose, and sectioned in 10 μm using a cryostat.

### BN-PAGE, SDS-PAGE, and immunoblotting

For BN-PAGE, muscle samples were homogenized by sonication in native PAGE sample buffer containing 1% dodecyl-B-D-maltoside. Homogenates were centrifuged at 12,400*g* for 10 minutes at 4°C. Supernatants were collected and protein concentrations were determined by DC assay (Bio-Rad). Homogenates (5 μg of protein each well) were mixed with 0.5% Coomassie G250 and subjected to 3%–12% Bis-Tris gel electrophoresis with native PAGE running buffer according to the manufacturer’s instructions (Novex). Proteins were then transferred onto PVDF membranes and fixed in 8% acetic acid. Membranes were rinsed with methanol to remove background dye.

For SDS-PAGE, muscle samples were homogenized by sonication in lysis buffer. Homogenates were centrifuged at 12,000 *g* for 10 minutes at 4°C. Supernatants were collected and protein concentrations were determined by DC assay (Bio-Rad). Homogenates (20 μg of protein each well) were electrophoresed in 10% SDS polyacrylamide gel. Proteins were transferred to PVDF membranes.

Next, membranes from BN-PAGE and SDS-PAGE were subjected to immunoblotting. Nonspecific bindings of the blots were blocked with 5% nonfat dry milk in Tris-buffered saline–Tween-20 (TBST). Blots were probed with rabbit anti–mouse AQP4 antibody (1:1,000; catalog A5971, Sigma-Aldrich) at 4°C overnight, washed with TBST, and incubated with HRP-conjugated anti-rabbit antibody (1:5,000; catalog P0448, Dako). After washing with TBST, signals were visualized by enhanced chemiluminescence (Advansta) using a ChemiDoc Imaging system (Bio-Rad).

### Cell-based indirect immunofluorescence assay

Cell-based indirect immunofluorescence assay was performed as described previously with modifications (31). Human embryonic kidney (HEK) 293 cells (ATCC) were seeded onto 8-well chamber slides at 10,000 cells per well and cultured in DMEM in 5% CO_2_ at 37°C. They were transfected with pAQP4 using Lipofectamine 3000 (Invitrogen) according to the manufacturer’s instructions. Cells were fixed with 4% paraformaldehyde, washed with PBS, and blocked with 1% BSA in PBS. Sera of mice were diluted at 1:10 with PBS containing 1% BSA. Commercially available rabbit anti–mouse AQP4 antibody (catalog A5971, Sigma-Aldrich) was diluted at 1:500 and used as positive control. To remove nonspecific antibodies, diluted sera were incubated with rat liver powder at room temperature for 1 hour and then centrifuged at 16,900*g* for 15 minutes. Next, 100 μL of the diluted sera were added to each well and incubated at 4°C overnight. After washing with PBS, cells were incubated with either Alexa Fluor 488–conjugated anti-mouse IgG (for samples) (catalog A21202) or anti-rabbit IgG (for positive control) (catalog A11070) secondary antibody (all from Thermo Fisher Scientific) at room temperature for 1 hour. Cells were coverslipped with antifade reagent containing DAPI (Thermo Fisher Scientific).

### Immunostaining and histochemistry

Cryosections of tibialis anterior muscles were incubated with rabbit anti–mouse AQP4 (1:200; catalog A5971, Sigma-Aldrich) and mouse anti-DDK (FLAG, 1:500; catalog TA50011-100, OriGene) at 4°C overnight. Cryosections of spinal cords and optic nerves were incubated with the following primary antibodies at 4°C overnight: rabbit anti–zonula occludens 1 (anti–ZO-1; 1:200; catalog AB216880, Abcam), goat anti–mouse IgG (1:100; catalog AB6708, Abcam), rabbit anti-AQP4 (1:200; catalog A5971, Sigma-Aldrich), mouse anti–glial fibrillary acidic protein (anti-GFAP; 1:200; catalog SC33673, Santa Cruz Biotechnology), rabbit anti–oligodendrocyte transcription factor 2 (anti-Olig2; 1:200; catalog AB9610, Millipore), goat anti–myelin basic protein (anti-MBP; 1:200; catalog SC13914, Santa Cruz Biotechnology), rabbit anti–neurofilament-heavy (anti–NF-H; 1:400; catalog N4142, Sigma-Aldrich), rabbit anti–ionized calcium–binding adapter molecule 1 (anti–Iba-1; 1:200; catalog 019-19741, Wako), rabbit anti–terminal complement complex (anti–C5b-9; 1:200; catalog AB55811, Abcam), rat anti–lymphocyte antigen 6 complex locus G6D (anti-Ly6G; 1:400; catalog AB2557, Abcam), rat anti–Siglec-F (1:50; catalog 552125, BD Biosciences), mouse anti–cluster of differentiation 68 (anti-CD68; 1:200; catalog YM3161, Immunoway), and rat anti-F4/80 (1:200; catalog AB6640, Abcam). Sections were then incubated with the appropriate Alexa Fluor–conjugated secondary antibodies (Thermo Fisher Scientific) at room temperature for 1 hour. Neuronal cells were visualized using blue fluorescent Nissl stain (NeuroTrace, Thermo Fisher Scientific). Sections were coverslipped with antifade reagent containing DAPI (Thermo Fisher Scientific). Selected sections were stained with H&E and Luxol fast blue using standard procedures.

### Microscopy

All images were captured using the same microscope (Eclipse Ni, Nikon) and digitized with SPOT software 5.0 (Diagnostic Instruments). Signal intensity was quantified using ImageJ software (version 1.53e, NIH).

### ELISA

Titer of AQP4 antibodies in mouse sera was determined using mouse anti-AQP4 competitive ELISA kit (catalog MBS746880, MyBioSource) according to the manufacturer’s instructions. Briefly, 96-well plates were precoated with AQP4. Sera from mice were added at serial dilutions from 1:10 to 1:10,000. Standards and samples were incubated with anti-AQP–HRP conjugate that competed for AQP4 antigen binding sites. After washing, color was developed with HRP substrate, and optical density was measured at 450 nm using a microplate reader (CLARIOstar, BMG Labtech).

Concentrations of TNF-α, IL-1β, and IL-6 in spinal cord homogenates were assessed using mouse sandwich ELISA kits (TNF-α, catalog ELM-TNFa-CL-1; IL-1β, catalog ELM-IL1b-CL-1; IL-6, catalog ELM-IL6-CL-1; all from RayBiotech) according to the manufacturer’s protocols. Briefly, standards and samples were added into 96-well plates precoated with anti–TNF-α, anti–IL-1β, or anti–IL-6 antibodies. After incubation, wells were washed, and biotinylated antibodies for mouse TNF-α, IL-1β, and IL-6 were added accordingly. After washing, HRP-conjugated streptavidin was added to wells; then wells were washed again and TMB substrate was added for color development. The optical density of each well was measured at 450 nm using a microplate reader (CLARIOstar, BMG Labtech). Concentrations (pg/mL) of the cytokines were normalized with the total protein content of the samples (pg/mg protein).

### Flow cytometry

#### Humans.

Blood samples from HCs and patients with NMOSD were collected in EDTA tubes. PBMCs were isolated by dilution of blood with RPMI 1640 medium (1:1) and layered over Ficoll-Paque PLUS density gradient medium (Cytiva) according to the manufacturer’s instructions. PBMCs were incubated with an antibody cocktail containing anti–human CD4–FITC (catalog 556615), anti–human CXCR5–PerCP-Cy5.5 (catalog 562781), and anti–human PD-1–PE (catalog 560908) (all from BD Biosciences) at 4°C for 30 minutes. Data were acquired using a CytoFLEX flow cytometer (Beckman Coulter) and analyzed using FlowJo software (version 10.8, FlowJo).

#### Mice.

Single-cell suspensions from the spleens of mice were isolated with 40 μm cell strainers. Cells were incubated with antibody cocktails containing anti–mouse CD16/CD32 Fc block (catalog 553142, BD Biosciences) at 4°C for 30 minutes. The antibodies were anti–mouse CD4–FITC (catalog 553729), anti–mouse CXCR5–PE-Cy7 (catalog 560671), anti–mouse PD-1–PE (catalog 551892), anti–mouse IFN-γ–PE (catalog 562020), anti–mouse IL-17A–PE (catalog 561020), anti–mouse CD19–V450 (catalog 560375), anti–mouse CD80–APC (catalog 560016), anti–mouse PD-L2–PE (catalog 557796), anti–mouse CD138–PE (catalog 561070), and anti–mouse TACI–Alexa Fluor 647 (catalog 558453) (all from BD Biosciences). Data were acquired using a CytoFLEX flow cytometer (Beckman Coulter) and analyzed using FlowJo software (version 10.8, FlowJo).

### Statistics

Differences between groups were compared by 1-way ANOVA with post hoc Tukey’s test. Differences between 2 groups were determined by Student’s 2-tailed *t* test. Calculations were performed using Prism 6 (GraphPad Software Inc.). Data are shown as the mean ± SEM. *P* values of less than 0.05 were considered significant.

### Study approval

The human study was approved by The University of Hong Kong/Hospital Authority Hong Kong West Cluster Institutional Review Board, Hong Kong. All animal experiments were approved by the Committee on the Use of Live Animals in Teaching and Research, The University of Hong Kong.

## Author contributions

LWY and KHC conceived and designed the study. LWY, OKFM, and JSCK performed the experiments. EYYC contributed to the experiment on *Aqp4^–/–^* mice. KXY organized patient visits and collected blood samples. LWY analyzed the data and wrote the manuscript. KHC gave comments on the manuscript. All authors revised and approved the manuscript.

## Supplementary Material

Supplemental data

## Version 1 01/17/2023

In-Press Preview

## Version 2 02/22/2023

Electronic publication
