# Supplementary material for: T follicular helper cells contribute to pathophysiology in a model of neuromyelitis optica spectrum disorders
Source: JCI Insight. 2023 Feb 22;8(4):e161003. doi: 10.1172/jci.insight.161003 (PMC9977492; doi:10.1172/jci.insight.161003)
Supplement: Supplemental data [file jciinsight-8-161003-s156.pdf]

**Characteristics**	**HC**	**NMOSD**
n	4	6
Mean age in years (Range)	40.8 (31-54)	47.7 (27-64)
Male, *n* (%)	1 (25)	2 (33.3)
Female, *n* (%)	3 (75)	4 (66.7)
AQP4-IgG seropositive, *n* (%)	-	5 (83.3)
Mean disease duration in years (Range)	-	6.3 (2-13)
Rituximab, *n* (%)	-	2 (33.3)
Mycophenolate mofetil, *n* (%)	-	3 (50)
Mycophenolate mofetil + cyclosporin A, *n* (%)	-	1 (16.7)
Mean EDSS (Range)	-	2.5 (1-4)
Relapsing-remitting disease, *n* (%)	-	6 (100)
Relapse in the last 6 months, *n* (%)	-	0 (0)

Supplemental Table 1. Demographics of HC and NMOSD patients. HC, healthy controls; NMOSD, neuromyelitis optica spectrum disorders; EDSS, expanded disability status scale.

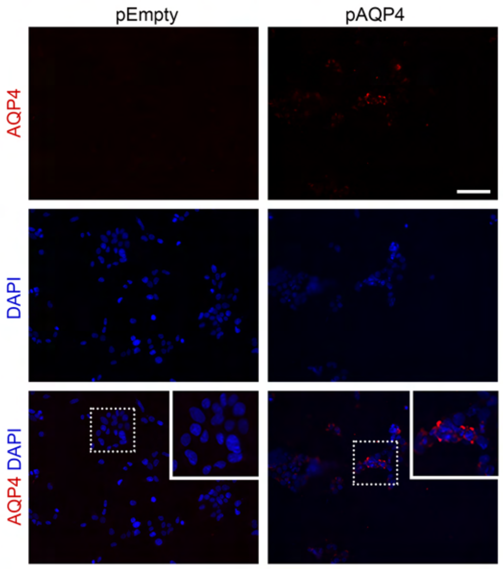

Supplemental Figure 1. AQP4 expression on the membrane of HEK293 cells. HEK293 cells were transfected with a plasmid encoding the M23 isoform of AQP4 (pAQP4) or an empty vector control (pEmpty). Immunostaining for AQP4 revealed a discontinuous and punctate distribution of AQP4 immunoreactivity suggestive of the expression of orthogonal array particles on the membrane of pAQP4 transfected cells, but not on that of control. Nuclei were counterstained with DAPI. Scale bar, 50 µm.

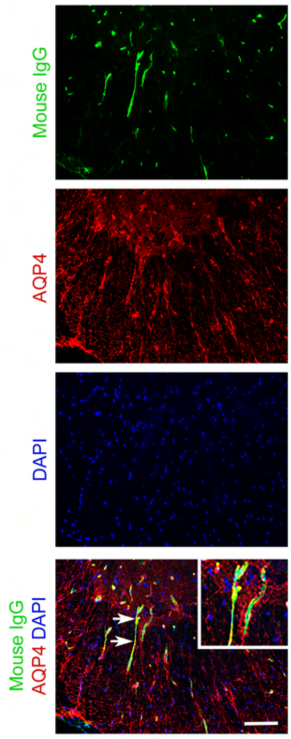

Supplemental Figure 2. Autoantibodies generated by immunization target AQP4 in the spinal cord. Co-immunostaining for mouse IgG and AQP4 in the spinal cord of pAQP4(C/P+) mice. Images are representative photomicrographs showing the ventrolateral white matter of cervical spinal cord cross sections from 5 mice per group. Nuclei were counterstained with DAPI. Scale bar, 50 µm.

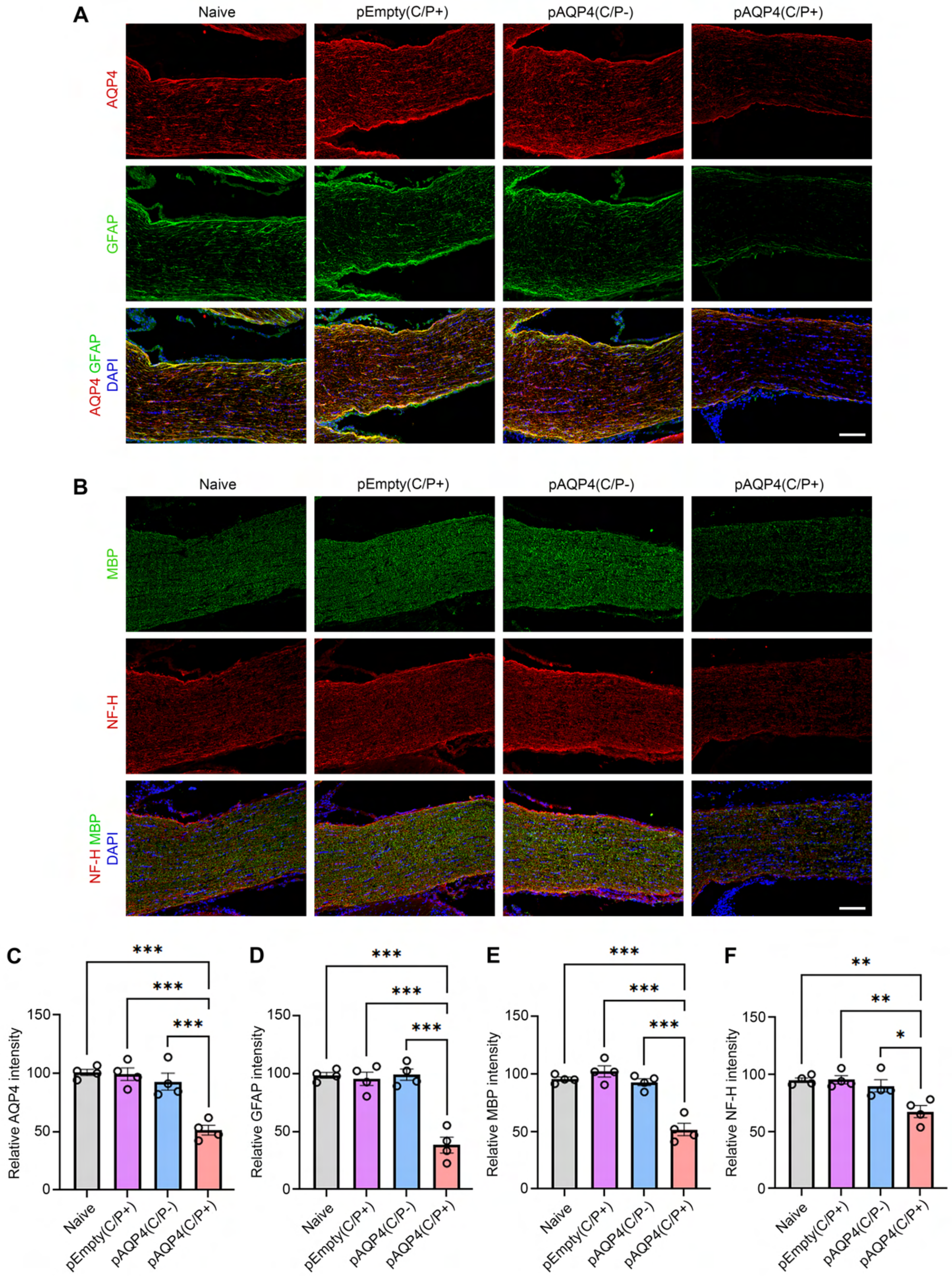

Supplemental Figure 3. Astrocytopathy, demyelination and axonal loss in the optic nerve after AQP4 immunization. (**A**) Co-immunostaining for AQP4 and GFAP in the optic nerve of naïve, pEmpty(C/P+), pAQP4(C/P-) and pAQP4(C/P+) mice. (**B**) Co-

immunostaining for MBP and NF-H in the optic nerve. (**C**-**F**) Respective quantification of AQP4, GFAP, MBP, and NF-H immunofluorescence intensities. Images are representative photomicrographs showing longitudinal sections of the optic nerve near the optic chiasma from 4 mice per group. Nuclei were counterstained with DAPI. Scale bar, 50 μm. Data are mean ± SEM; n = 4 per group. ***P < 0.001, **P < 0.01, *P < 0.05, one-way ANOVA with post hoc Tukey's test.

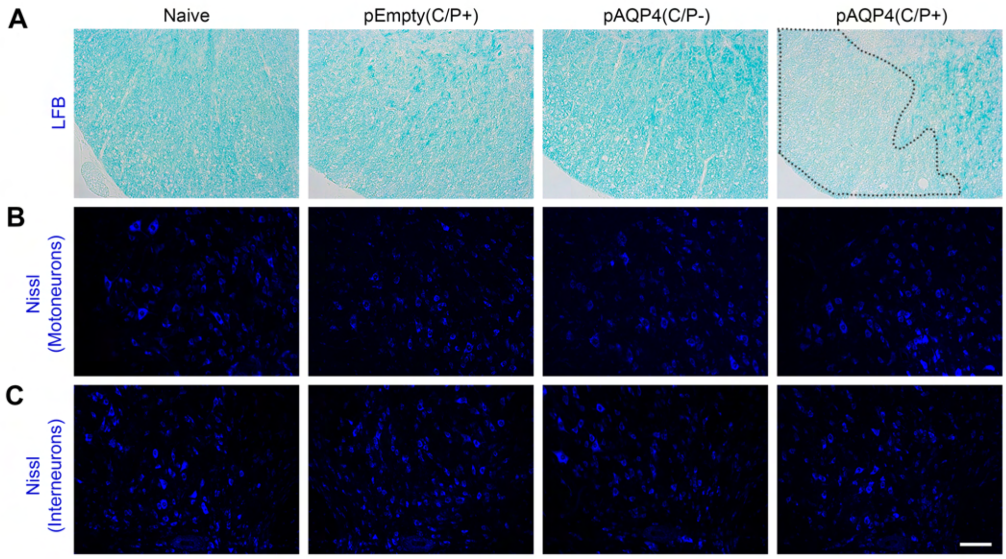

Supplemental Figure 4. Demyelination without neuronal loss after AQP4 immunization. (**A**) Luxol fast blue staining for myelin in the ventrolateral white matter of the cervical spinal cord. Dotted line demarcates the demyelinated spinal cord white matter. (**B**-**C**) Fluorescent Nissl staining for motoneurons in the ventral horn (**B**) and interneurons near the central canal (**C**) of the spinal cord. Images are representatives of 5 mice per group. Scale bar, 50 µm.

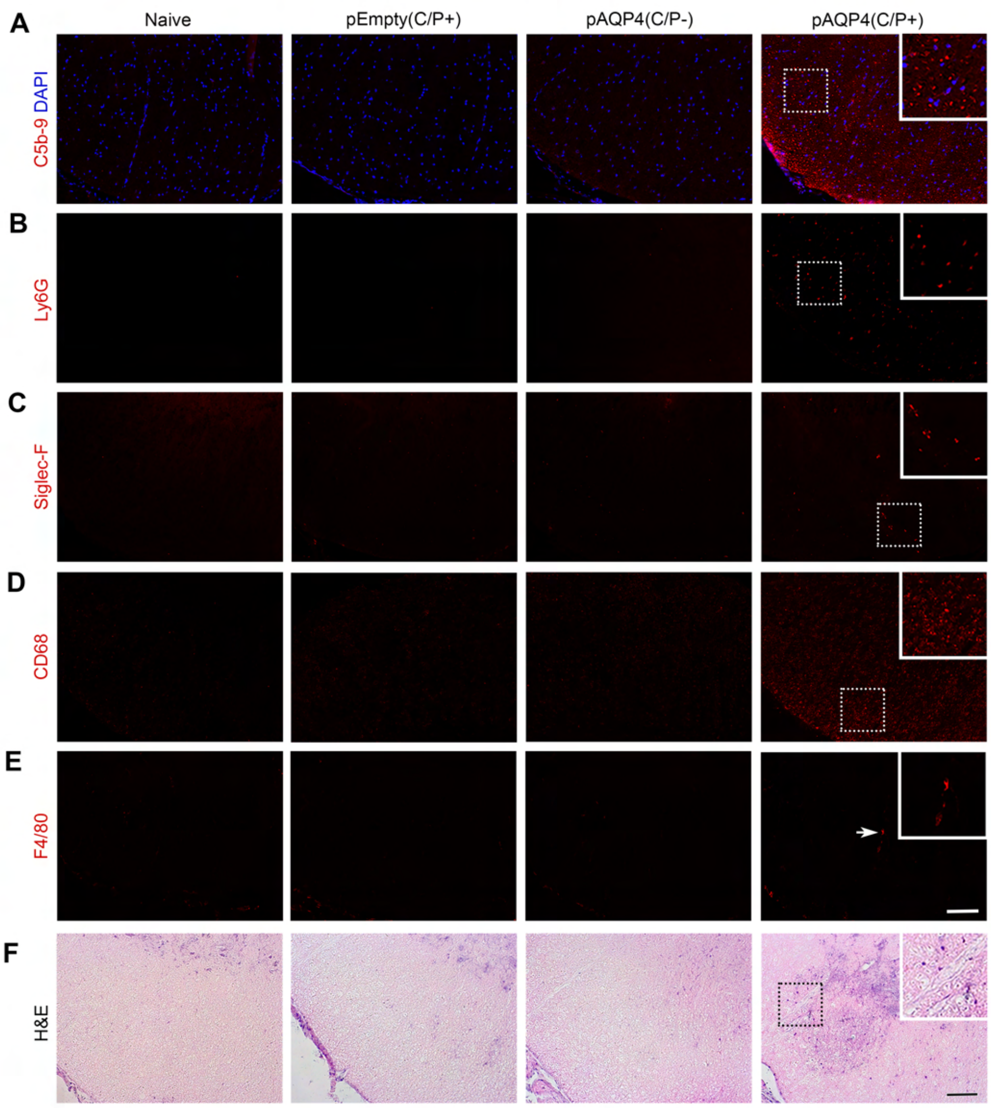

Supplemental Figure 5. Deposition of terminal complement complex and infiltration of inflammatory cells in the spinal cord after AQP4 immunization. (**A-C**) Immunostaining for C5b-9 (terminal complement complex) (**A**), Ly6G (neutrophils) (**B**), and Siglec-F (eosinophils) (**C**) in the spinal cord of naïve, pEmpty(C/P+), pAQP4(C/P-) and pAQP4(C/P+) mice. (**D** and **E**) Immunostaining for CD68 (both brain-derived and infiltrated macrophages) (**D**) and F4/80 (infiltrated macrophages) (**E**). (**F**) H&E staining. Images are representative photomicrographs showing the ventrolateral white matter of cervical spinal cord cross sections from 5 mice per group. Insets are higher magnification photomicrographs. Scale bar, 50 µm.

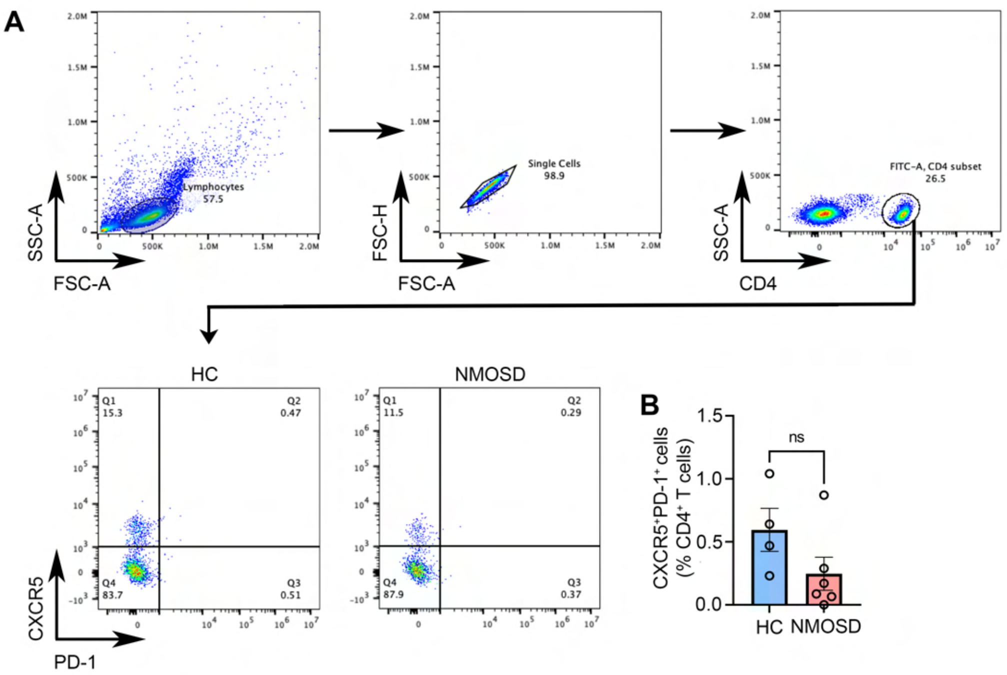

Supplemental Figure 6. Frequency of circulating Tfh cells in HC and treated NMOSD patients. (**A**) Upper panel, human Tfh-cell gating strategy. Sideward scatter-A (SSC-A) and forward scatter-A (FSC-A) were used to gate lymphocytes. FSC-H and FSC-A were used to gate single cells. Tfh cells were defined as $CD4^+CXCR5^+PD\text{-}1^+$ lymphocytes. Lower panel, representative flow cytometry plots of $CXCR5^+PD\text{-}1^+$ Tfh cells in the PBMC of treated HC and NMOSD patients, pre-gated on $CD4^+$ T cells. (**B**) Percentage of $CXCR5^+PD\text{-}1^+$ cells in $CD4^+$ cells of treated HC and NMOSD patients. Data are mean ± SEM; n = 4 to 5 per group. ns, non-significant, Student's *t* test.

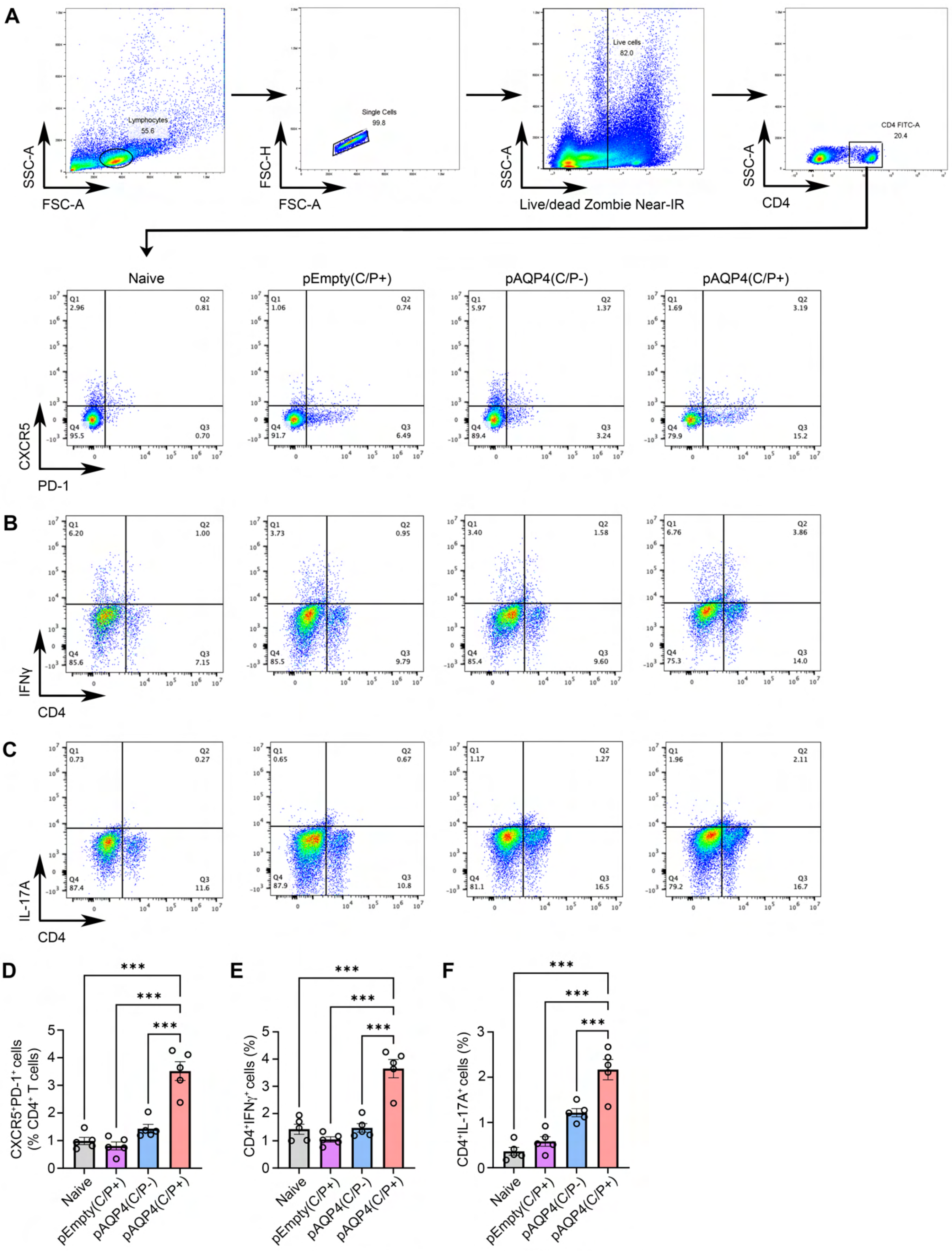
A
SSC-A
FSC-A
Lymphocytes
55.6
FSC-H
FSC-A
Single Cells
99.8
SSC-A
Live/dead Zombie Near-IR
Live cells
82.0
SSC-A
CD4
CD4 FITC-A
20.4
Naive
pEmpty(C/P+)
pAQP4(C/P-)
pAQP4(C/P+)
CXCR5
PD-1
B
IFNγ
CD4
C
IL-17A
CD4
D
CXCR5+PD-1+ cells (% CD4+ T cells)
E
CD4+IFN-γ+ cells (%)
F
CD4+IL-17A+ cells (%)
***
Naive
pEmpty(C/P+)
pAQP4(C/P-)
pAQP4(C/P+)

Supplemental Figure 7. AQP4 immunization induces the expansion of Tfh, Th1 and Th17 cells. (**A**) Upper panel, mouse Tfh-cell gating strategy. Sideward scatter-A (SSC-A) and forward scatter-A (FSC-A) were used to gate lymphocytes. FSC-H and FSC-A were used to gate single cells. SSC-A and Live/Dead Zombie were used to gate live cells. Tfh cells were defined as $CD4^+CXCR5^+PD\text{-}1^+$ lymphocytes. Lower panel, representative flow cytometry plots of $CXCR5^+PD\text{-}1^+$ Tfh cells in the spleen of naïve, pEmpty(C/P+), pAQP4(C/P-) and pAQP4(C/P+) mice, pre-gated on $CD4^+$ T cells. (**B**) Representative flow cytometry plots of $CD4^+IFN\gamma^+$ Th1 cells. (**C**) Representative flow cytometry plots of $CD4^+IL\text{-}17A^+$ Th17 cells. (**D-F**) Flow data are quantified for all groups. Data are mean ± SEM; n = 5 per group. ***P < 0.001, one-way ANOVA with post hoc Tukey's test.

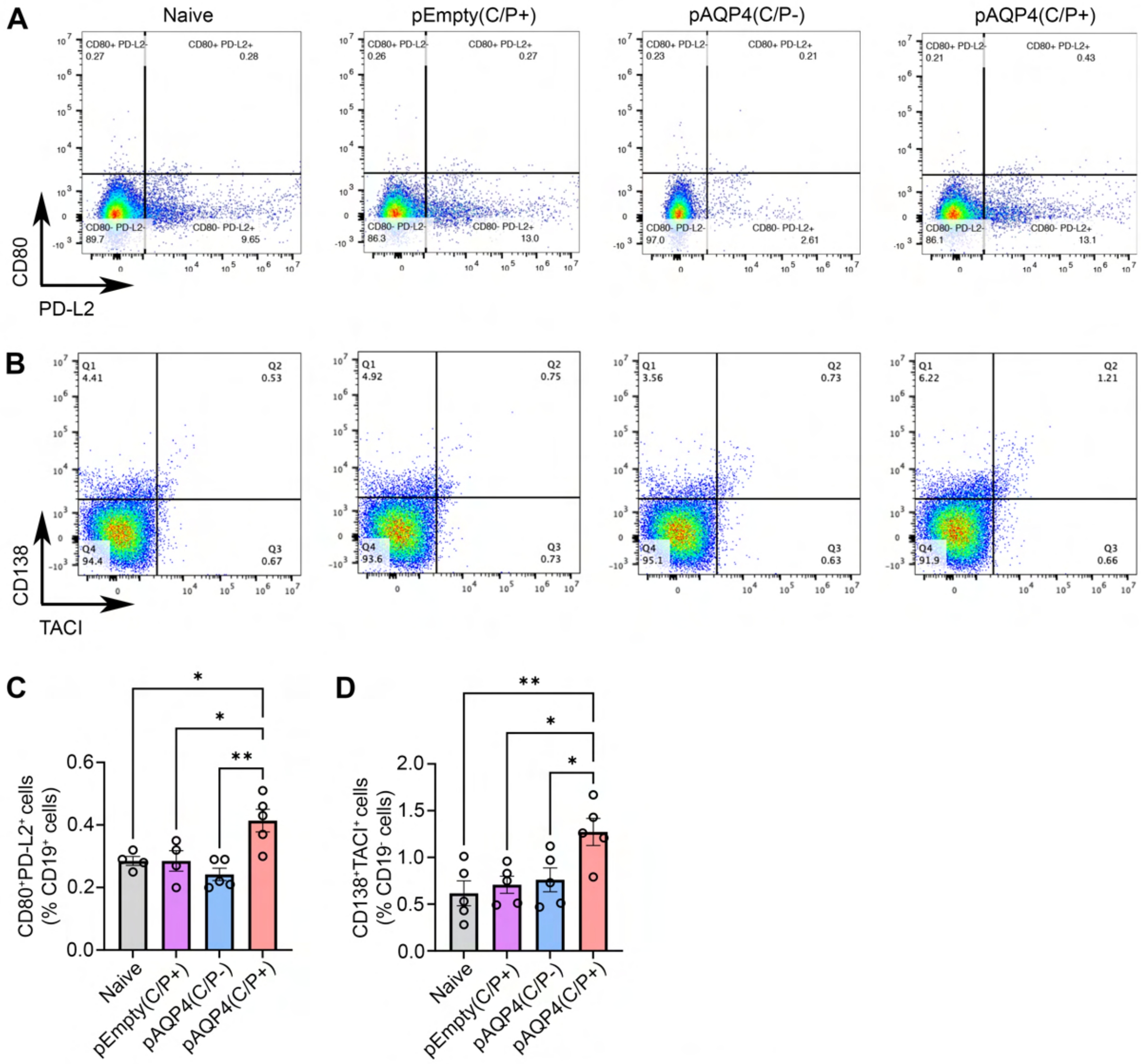

Supplemental Figure 8. AQP4 immunization induces the expansion of memory B cells and plasma cells. Memory B cells and plasma cells were defined as $CD80^+PD\text{-}L2^+$ and $CD138^+TACI^+$ lymphocytes, respectively. (**A**) Representative flow cytometry plots of $CD80^+PD\text{-}L2^+$ memory B cells in the spleen of naïve, pEmpty(C/P+), pAQP4(C/P-) and pAQP4(C/P+) mice. (**B**) Representative flow cytometry plots of $CD138^+TACI^+$ plasma cells. (**C and D**) Flow data are quantified for all groups. Data are mean ± SEM; n = 4 to 5 per group. $^{**}P < 0.01$, $^{*}P < 0.05$, one-way ANOVA with post hoc Tukey's test.
